# Combine photosynthetic characteristics and leaf hyperspectral reflectance for early detection of water stress

**DOI:** 10.3389/fpls.2025.1520304

**Published:** 2025-04-09

**Authors:** Linbao Li, Guiyun Huang, Jinhua Wu, Yunchao Yu, Guangxin Zhang, Yang Su, Xiongying Wang, Huiyuan Chen, Yeqing Wang, Di Wu

**Affiliations:** ^1^ Yangtze River Biodiversity Research Centre, China Three Gorges Corporation, Wuhan, China; ^2^ China Three Gorges Corporation, Hubei Key Laboratory of Rare Resource Plants in Three Gorges Reservoir Area, Yichang, China; ^3^ National Engineering Research Center of Eco-Environment Protection for Yangtze River Economic Belt, China Three Gorges Corporation, Wuhan, China; ^4^ Wufeng Houhe National Nature Reserver, Hubei Forestry Bureau, Yichang, China

**Keywords:** chlorophyll fluorescence, hyperspectral reflectance, leaf chlorophyll content, machine learning algorithms, *Rhamnus leptophylla*, water stress

## Abstract

Advanced techniques capable of early and non-destructive detection of the impacts of water stress on trees and estimation of the underlying photosynthetic capacities on larger scale are necessary to meet the challenges of limiting plant growth and ecological protection caused by drought. We tested influence of continuous water stress on photosynthetic traits including Leaf Chlorophyll content (LCC) and Chlorophyll Fluorescence (ChlF) and combined hyperspectral reflectance as a high-throughput approach for early and non-destructive assessment of LCC and ChlF traits in *Rhamnus leptophylla* trees. LCC and ChlF parameters (NPQ, Fv’/Fm’, ETR, ETRmax, Fm’, qL, qP, Y(II) were measured alongside leaf hyperspectral reflectance from *Rhamnus leptophylla* suffering from constant drought during water stress. Water stress caused NPQ, Fv’/Fm’, ETRmax, Fm’, qL, qP, Y(II) and ETR continuous decline throughout the entire drought period. ChlF was more sensitive to drought monitoring than LCC. The original reflectance spectra and hyperspectral vegetation indices (SVIs) showed a strong correlation with LCC and ChlF. Reflectance in 540-560nm and 750-1100nm and selected SVI such as Simple Ratio (SR)752/690 can track drought responses effectively before leaves showed drought symptoms. Multivariate Linear Regression (MLR) and three machine learning algorithms, namely Random Forest (RF), Support Vector Machine (SVM), and K-Nearest Neighbor (KNN) were employed to develop models for estimating LCC and ChlF parameters. RF provided the best estimation accuracy for LCC compared to MLR, KNN and SVM, achieving an R^2^ value of 0.895 for all LCC samples. The canopy layer significantly influenced the estimation accuracy of LCC, with the middle layer yielding the highest R^2^ value. RF also demonstrated superior performance compared to MLR, KNN and SVM for estimating NPQ, Fv’/Fm’, ETRmax, Fm’, qL, qP, Y(II) and ETR, achieving R^2^ value of 0.854 for NPQ, 0.610 for Fv’/Fm’, 0.878 for ETRmax, 0.676 for Fm’, 0.604 for qL, 0.731 for qP, 0.879 for Y(II), and 0.740 for ETR. Our results indicate that photosynthetic traits combined hyperspectral reflectance can monitor the effect of drought on trees effectively with significant potential for monitoring drought over large areas.

## 1 Introduction

Global warming is a significant consequence of human activities, primarily driven by the overuse of fossil fuels, which has led to an increased concentration of greenhouse gases in the atmosphere. This rise in greenhouse gas levels is responsible for the increasing average surface temperature of the Earth (Al-Ghussain, 2019). Furthermore, it enhances evaporation rates and decreases soil moisture content (Samaniego et al., 2018). Consequently, climate change may exacerbate drought conditions, leading to more rapid onset, increased intensity, and prolonged duration of drought events (Trenberth et al., 2014). Water stress often leads to plant dehydration, disrupting the ability of plant cells to maintain normal water concentration levels for their physiological activities (Porporato et al., 2001; Rad et al., 2022). Therefore, water stress is one of the most important abiotic stress factors limiting plant growth and agricultural productivity (Chaves et al., 2002; Gerhards et al., 2019; Alagoz et al., 2023; Samadi et al., 2024). Photosynthesis is an important physiological activity in the growth process of green plants, which is sensitives to soil drought. And water stress often leads to low net photosynthetic rates (Xiao et al., 2019). Traditional water deficit monitoring was achieved by measuring soil moisture content by using soil moisture measuring instrument quickly. Loose voids in the soil can result to delayed and inaccurate monitoring drought for plants. It may be the quickest and most direct way to detect drought through leaf physiology such as photosynthetic traits.

Chlorophyll and chlorophyll fluorescence was key traits allowing for the assessment of photosynthetic capacity and adaptability of plants. Chlorophyll is a key pigment in photosynthesis which participating in acquisition and conversion of light energy for providing essential biochemical energy for Calvin–Benson cycle (Evans, 1989; Peng et al., 2017). leaf chlorophyll content (LCC) and Chlorophyll fluorescence (ChlF) serves as a natural indicator for assessing the photosynthetic capacity of leaves, reflecting the efficiency of photosynthesis and the allocation of photosynthetic products in plants (Linn et al., 2021; Song et al., 2024). And it is also a non-invasive tool for assessing plant stress and adaptation mechanisms under drought conditions (Salvatori et al., 2016). The examination of variations in plant chlorophyll fluorescence enhances comprehension of the efficacy of light energy absorption, conversion, and utilization within the plant photosynthetic system at a microscopic scale (Pleban et al., 2020).

Plants can be irreversibly affected before visible symptoms of water stress appear (Yordanov et al., 2003). Compared to traditional field measurements, remote sensing can provide timely and reliable information about the plant physiology with a cost-effective way (Bouman et al., 1996). Hyperspectral data are ranging from the visible over the near infrared to the intermediate infrared and can provide spectral features regarding differences in leaf metabolism, structure, and physiological and chemical traits with non-destructive ways at different scales (Yendrek et al., 2017; Sonobe et al., 2020a, 2020b; Streher et al., 2020; Zhou et al., 2021). Hyperspectral Reflectance have been used for early detecting scab induced stress in apple leaves, water stress in citrus and grapevine, salinity stress in Myrica cerifera, Hydrogen Peroxide in Sorghum before symptoms become visible to the naked eye (Naumann, 2008; Maimaitiyiming et al., 2017; Zhou et al., 2021; Song et al., 2023). However, the mechanisms linking spectra reflectance to plant functional traits are not always clear, because application of hyperspectral spectra to assess plant function or physiology is often complex (Gerhards et al., 2019). How to select stable spectral parameters or vegetation index which can characterize physiological and biochemical changes of plants in environmental stress is still a big challenge.

*Rhamnus leptophylla*, a common shrub or small tree in the Three Gorges area, plays a key role in assessing and monitoring drought stress, which determines its potential for stabilizing side slopes in the fluctuation zone. The selection of *Rhamnus leptophylla* was based on its hypothesized adaptation strategies and potential tolerance to drought stress. It remains unclear whether it is feasible to conduct early drought diagnosis of *Rhamnus leptophylla* by combining photosynthetic parameters and hyperspectral data. In this study, we measured the field photosynthetic traits including LCC, ChlF parameters and corresponding hyperspectral reflectance over progressive water stress and aimed to (1) explore the effect of water stress to photosynthetic capacities; (2) What is the variation in the leaf reflectance in continuous water stress? What is the key hyperspectral information of *Rhamnus leptophylla* leaves responding to water stress? (3) compare the potential of various algorithms, including multivariate linear regression and machine learning techniques, for estimating LCC and ChlF under varying water stress.

## 2 Materials and methods

### 2.1 Experimental design

**Figure 1:**
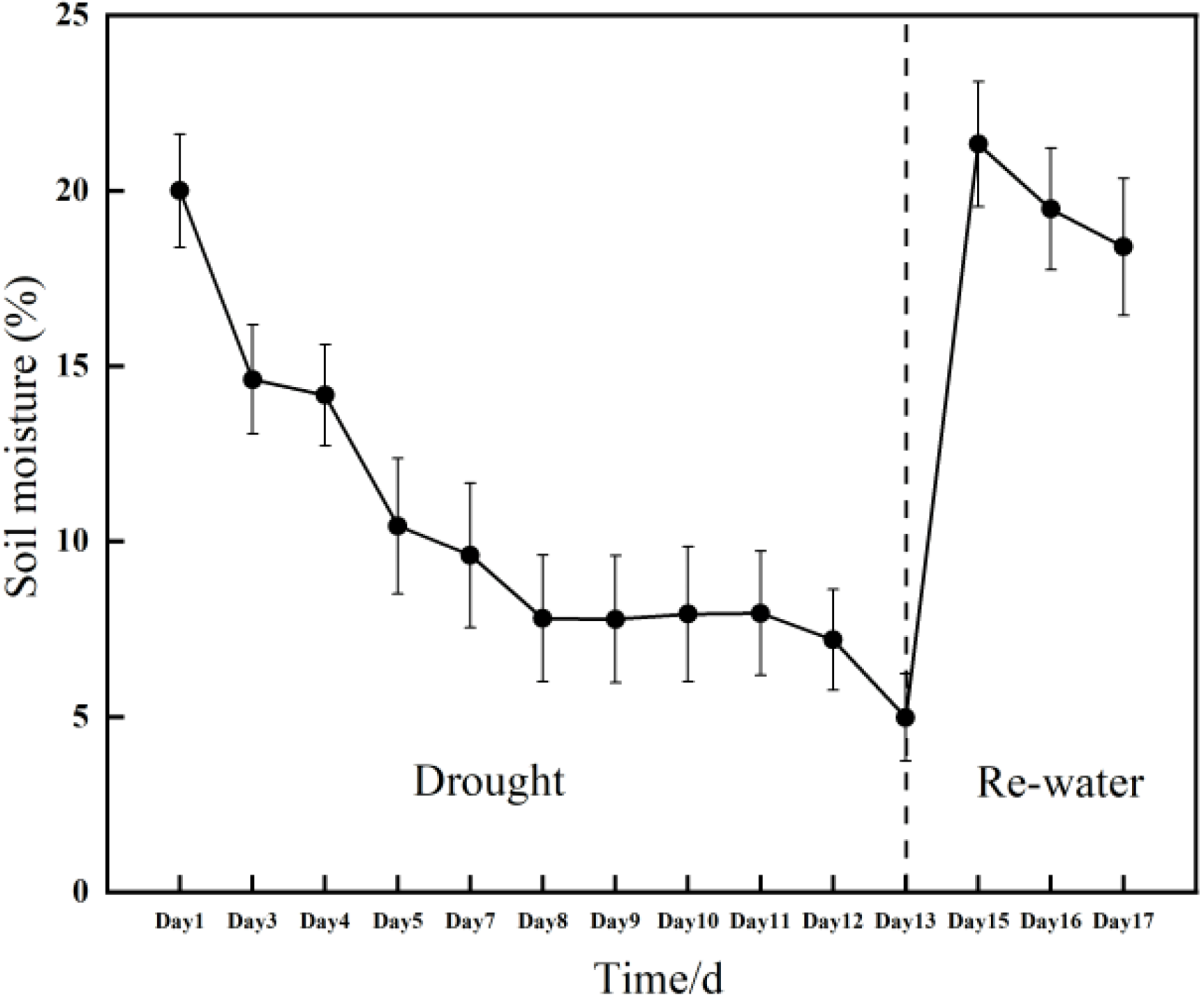
Changes in soil moisture during drought conditions and subsequent re-watering of the *Rhamnus leptophylla* trees.

This experiment was conducted using *Rhamnus leptophylla* as selected planted material at the greenhouse facility of special plant germplasm resource garden of the Institute of Endangered Plants of the Three Gorges Reservoir. The region experiences maximum and minimum temperatures of 44°C and -2.5°C, respectively, with an annual average temperature of 18°C. The relative air humidity is 77% and 75% (1000 to 1025 mm) of annual precipitation take place from April to September (Zeng et al., 2018). Nine three-year-old plum plant was used, and their height ranged from 2 m to 2.5 m. They were planted in 40 cm plastic pots containing potting mix of commercial substrate and perlite. All the trees were exposed to natural conditions in the experiment during from 15 July to 31 July of 2024 (day 1 to day 17). All the trees were watered to field water capacity in 15 July (onset of the water stress treatment) and not irrigated until 27 July (13 days). Then we re-watered all the *Rhamnus leptophylla* trees in 28 July (day 14). The change of soil water content with time was illustrated in **Figure 1**.

Leaf chlorophyll content and spectral-related parameters were measured almost daily from July 15 (day 1) to July 31 (day 17) on nine leaves from the upper, middle, and lower layers of nine trees. We also measured the chlorophyll fluorescence parameters of three leaves from the upper, middle, and lower layers for each plant, along with the corresponding hyperspectral reflectance, from July 15 (day 1) to July 31 (day 17).

### 2.2 Hyperspectral data acquisition

A portable ASD FieldSpecHH spectrometer (325-1075 nm range, 1 nm resolution; ASD Inc., Boulder, United States) was used to measure the spectral reflectance of *Rhamnus leptophylla* leaves. Each leaf was measured ten times for getting mean value as the representative reflectance of each leaf. When the measurement was conducted, leaf veins should be kept away for obtaining the reflectance of true leaf tissue.

### 2.3 Determination of leaf chlorophyll content

LCC was measured by using a handheld chlorophyll meter (SPAD-502Plus, Konica Minolta, Tokyo, Japan) in the field. The chlorophyll meter primarily utilizes leaf transmittance within the central band of 650 to 940 nm to determine chlorophyll content, and SPAD values can more accurately reflect changes in leaf greenness (Ta et al., 2021). Each sample value was obtained from the same location as where spectral data were obtained. For every sample leaf, three measurements were taken, and these values were then averaged to derive the representative SPAD value for the leaves.

### 2.4 Measurement of chlorophyll fluorescence parameters

The chlorophyll fluorescence of the same leaves was analyzed with a MINI-PAM-II fluorometer (Imaging PAM, Walz, Effeltrich, Germany) by User Manual and a previously described method (Gong et al., 2019). The photochemical efficiency of PSII in the light (Fv’/Fm’) was measured directly under light without dark adaptation. The nonphotochemical quenching coefficient (NPQ) were calculated based on dark- and light-adapted fluorescence measurements. Maximum electron transfer rate (ETRmax) was obtained by calculating the fitting curve between a series of photosynthetically active radiation (PAR) and ETR. In addition, we also obtained maximal fluorescence in the presence of NPQ (Fm’), yield of quantum efficiency(Y(II)), apparent photosynthetic electron transport rate (ETR) and two photochemical quenching coefficient (qP and qL).

### 2.5 Extraction of spectral parameters

In total, 56 vegetation indices (VIs), 4 three-edge parameters (TEPs), and first-order differential spectrum (FODS) were selected for LCC estimations which are presented in **Table 1**. The indices included some traditional and popular vegetation indices (VIs), such as normalized difference vegetation index (e.g., NDVI), simple ratio indices (e.g., SR), photochemical vegetation index (e.g., PRI) and ratio vegetation indices (e.g., RVI). VIs simplifies the interpretation of complicated vegetation reflection patterns by establishing indirect connections with plant physiological and structural characteristics (Gerhards et al., 2019; Zhou et al., 2021). FODS and TEPs can reflect the spectral attributes of green vegetation well and exhibits sensitivity to variations in LCC (Li et al., 2023). All data processing and spectral calculations were conducted using the Python programming language v3.10.

**Table 1:** The 61 selected spectral parameters examined in this study, along with their band-specific formulations and corresponding principal references.

NO.	Name	Explanation	Reference
1	Anthocyanin Reflectance Index 1	ARI1 = 1/R550 − 1/R700	(Gitelson et al., 2001)
2	Anthocyanin Reflectance Index 2	ARI2 = R800**×**(1/R550 − 1/R700)	(Liang et al., 2016)
3	Green Normalized Difference Vegetation Index hyper 1	GNDVIhyper1 = (R750 − R550)/(R750 + R550)	(Liang et al., 2016)
4	Green Normalized Difference Vegetation Index hyper 2	GNDVIhyper2 = (R800 − R550)/(R800 + R550)	(Liang et al., 2016)
5	Modified Normalized Difference Vegetation Index	mNDVI705 = (R750 − R705)/(R750 + R705 − 2R445)	(Liang et al., 2016)
6	Modified simple ratio	mSR_705 = (R_750_ **−** R_445_)/(R_705_+R_445_)	(Sims and Gamon, 2002)
7	Canopy Chlorophyll Index	CCI = (R777 − R747)/R673	(Liang et al., 2016)
8	Vogelmann Index 2	VOG2 = (R_734_ − R_747_)/(R_715_ + R_726_)	(Liang et al., 2016)
9	Simple Ratio	SR = R_800_/R_680_	(Jordan, 1969)
10	Carter1	Carte1 = R695/R420	(Carter, 1994)
11	Carter2	Carte2 = R695/R760	(Carter, 1994)
12	Carter3	Carte3 = R605/R760	(Carter, 1994)
13	Carter4	Carte4 = R710/R760	(Carter, 1994)
14	Carter5	Carte5 = R695/R670	(Carter, 1994)
15	Photochemical vegetation index	PRI = (R_570_-R_531_)/(R_570_+R_531_)	(Yang et al., 2023)
16	Datt1	Datt1 = (R850 − R710)/(R850 − R680)	(Datt, 1999a)
17	Datt2	Datt2 = R850/R710	(Datt, 1999a)
18	Datt3	Datt3 = R754/R704	(Datt, 1999a)
19	Enhanced Vegetation Index	EVI = 2.5**×**((R800 − R670)/R800 − 6**×**R670 − 7.5**×**R475 + 1))	(Huete et al., 1994)
20	Modified Chlorophyll Absorption in Reflectance Index	MCARI = ((R700 − R670) − 0.2**×**(R700 − R550))(R700/R670)	(Daughtry et al., 2000)
21	Modified Triangular Vegetation Index 1	MTVI1 = 1.2**×**(1.2**×**(R800 − R550) − 2.5 × (R670 − R550))	(Haboudane et al., 2004)
22	Normalized Difference Cloud Index	NDCI = (R762 − R527)/(R762 + R527)	(Marshak et al., 2000)
23	Plant Senescence Reflectance Index	PSRI = (R678 − R500)/R750	(Merzlyak et al., 1999)
24	Renormalized Difference Vegetation Index	RDVI = (R_800_ − R_670_)/ $\sqrt{{\text{R}}_{800}+{\text{R}}_{670}}$	(Roujean and Breon, 1995)
25	Red-Edge Position Linear Interpolation	REP = 700 + 40**×**((R_670_ + R_780_)/2 − R_700_)/(R_740_ − R_700_)	(Clevers, 1994)
26	Spectral Polygon Vegetation Index 1	SPVI1 = 0.4**×**3.7**×**(R_800_ − R_670_) − 1.2**×**|R_530_ − R_670_|	(Vincini et al., 2005)
27	Simple Ratio Pigment Index	SRPI = R_430_/R_680_	(Penuelas et al., 1995)
28	Transformed Vegetation Index	TVl = 0.5×(120×(R_750_ **−** R_550_)) **−**200×(R_670_ **−** R_550_)	(Broge and Leblanc, 2001)
29	Simple Ratio 440/690	SR(440,690) = R_440_/R_690_	(Lichtenthaler et al., 1995)
30	Simple Ratio 700/670	SR(700,670) = R_700_/R_670_	(McMurtrey et al., 1994)
31	Simple Ratio 750/550	SR(750,550) = R_750_/R_550_	(McMurtrey et al., 1994)
32	Simple Ratio 750/700	SR(750,700) = R_750_/R_700_	(Gitelson and Merzlyak, 1997)
33	Simple Ratio 750/710	SR(750,710) = R_750_/R_710_	(Zarco-Tejada and Miller, 1999)
34	Simple Ratio 752/690	SR(752,690) = R_752_/R_690_	(Zarco-Tejada and Miller, 1999)
35	Simple Ratio 800/680	SR(800,680) = R_800_/R_680_	(Sims and Gamon, 2002)
36	Simple Ratio 735/720	SR(735,720) = R_735_/R_720_	(Zarco-Tejada and Miller, 1999)
37	Improved odds index	MSR = (R_800_/R_670_ **−** 1)/(R_800_/R_670_+1)	(Broge and Leblanc, 2001)
38	Transformed Chlorophyll Absorption Ratio	TCARI = 3**×**((R_700_ − R_670_) − 0.2**×**(R_700_ − R_550_)(R_700_/R_670_))	(Haboudane et al., 2002)
39	Optimized Soil Adjusted Vegetation Index	OSAVI = (1 + 0.16)**×**(R_800_ − R_670_)/(R_800_ + R_670_ + 0.16)	(Rondeaux et al., 1996)
40	Transformed Chlorophyll Absorption in Reflectance Index/Optimized Soil Adjusted Vegetation Index	TCARI/OSAVI = $\frac{3\times (({\text{R}}_{700}-{\text{R}}_{670})-0.2\times ({\text{R}}_{700}-{\text{R}}_{550})({\text{R}}_{700}/{\text{R}}_{670}))}{\left(1+0.16)\times ({\text{R}}_{800}-{\text{R}}_{670})/({\text{R}}_{800}+{\text{R}}_{670}+0.16\right)}$	(Liang et al., 2016)
41	Triangular Vegetation Index	TVI = 0.5**×**(120**×**(R_750_ − R_550_) – 200**×**(R_670_ − R_550_))	(Broge and Leblanc, 2001)
42	Leaf Chlorophyll Index	LCI = $\frac{\left|{\text{R}}_{850}|-|{\text{R}}_{710}\right|}{\left|{\text{R}}_{850}|-|{\text{R}}_{680}\right|}$	(Datt, 1999b)
43	Green carotenoid index	CAR_green = (1/R_510_-1/R_550_)×R_770_	(Gitelson et al., 2005)
44	Structure Intensive Pigment Index 1	SIPI1 = (R_800_ − R_445_)/(R_800_ − R_680_)	(Blackburn, 1998)
45	Structure Intensive Pigment Index 2	SIPI2 = (R_800_ − R_505_)/(R_800_ − R_690_)	(Blackburn, 1998)
46	Structure Intensive Pigment Index 3	SIPI3 = (R_800_ − R_470_)/(R_800_ − R_680_)	(Blackburn, 1998)
47	Red-Edge Ratio Vegetation Index	RERVI = R_840_/R_717_	(Gitelson et al., 2005)
48	Red-Edge Normalized Difference Vegetation Index	RENDVI = (R_840_ − R_717_)/(R_840_ + R_717_)	(Fitzgerald et al., 2010)
49	Red-edged vegetation stress index	RVSI = (R_712_ **−** R_670_)/2 **−** R_732_	(Devadas et al., 2009)
50	Green Ratio Vegetation Index	GRVI = R_840_/R_560_	(Gitelson et al., 2005)
	Greenness index	GI = R_554_/R_667_	(Yang et al., 2023)
51	MERIS Terrestrial Chlorophyll Index	MTCI = (R_753_ − R_708_)/(R_708_ − R_681_)	(Dash and Curran, 2004)
52	Chlorophyll Index Green	CI-green = (R_780_/R_550_) − 1	(Gitelson et al., 2006)
53	Normalized chlorophyll ratio index	NPCI = (R_680_ **−** R_630_)/(R_680_+R_630_)	(Chen, 1996)
54	Ratio Vegetation Index	RVI = R_765_/R_720_	(Jordan, 1969)
55	Colour content index	R_800_ = R_800_ **−** R_550_	(Yang et al., 2023)
56	FODS	First-order differential spectrum	(Li et al., 2019)
57	SDr	First-order differential spectral integration in the wavelength range of 680~760 nm	(Li et al., 2007)
58	SDb	First-order differential spectral integration in the wavelength range of 490~530 nm	(Li et al., 2007)
59	SDr/SDb	Ratio of the red edge area to the blue edge area	(Li et al., 2007)
60	(SDr − SDb)/(SDr + SDb)	Normalized value of the red edge area and the blue edge area	(Li et al., 2007)

R, r, and b represent spectral reflectance, red edge, and blue edge, respectively. NO.1~56, 57, and 58~61 was the VIs, FODS, and TEPs, respectively.

### 2.6 Data analysis

An analysis of variance (ANOVA) was conducted to examine the effects of drought and canopy layers on the leaf chlorophyll content (LCC) and Chlorophyll fluorescence parameters of *Rhamnus leptophylla* trees. *Post-hoc* multiple comparisons were performed using the least significant difference (LSD) method. ANOVA and LSD also were applied for selecting key hyperspectral SVIs. Three machine learning algorithms including K-Nearest Neighbor (KNN), Support Vector Machines (SVM) and Random Forest regression (RF), were applied to estimated LCC and ChlF parameters.

SVM employ a nonlinear kernel function to map input data into a high-dimensional feature space, enabling the representation of complex nonlinear patterns in a simplified manner (Mountrakis et al., 2011). For optimal SVR performance, a step involved the tuning of hyperparameters. The hyperparameters selected for tuning were the regularization parameter (C), and the kernel coefficient (g) for the three kernel functions: linear functions, radial basis kernel functions, and polynomial kernel functions. C and g were optimized within [10

KNN is a relatively simple method in which the estimation is predicted as a weighted average value with k spectrally nearest neighbors using a weighting method (Zhang et al., 2018). The KNN parameters were set as follows: the type of distance measures was set to Euclidean distance and Manhattan distance, the weighting functions were set to uniform, algorithm was set “auto”, and n_neighbors was set to [5,15,20,30,25] (Li et al., 2023).

RF is an ensemble machine learning technique that relies on decision trees. It constructs numerous small regression trees to make predictions (Boochs et al., 1990). The primary hyperparameters of RF model consist of the number of trees, maximum depth, min_samples_split, and min_samples_leaf. In this study, the hyperparameters for various photosynthetic parameters were defined as follows: the number of trees was set within a range of [50, 200], the maximum depth was configured as [None, 5], and both min_samples_split and min_samples_leaf were assigned a range of [1, 5].

We performed consecutive measurements across three layers (three leaves per layer) and over a period of 14 days, resulting in a large dataset suitable for model building. And the whole number of samples was 1134. The greenhouse measured data were randomly divided into training (80%) and testing (20%) data. To determine the relationship between the predicted and measured values, the overall model is evaluated in the graph including linear regression and a 1:1 dash-line. Ten - fold cross validation was applied for calculating RMSE to enhance the robustness. The predictive performance of each estimation model was evaluated using the coefficient of determination (R^2^), root mean square error (RMSE) and Bias, calculated as in the following equations:


(1) $${\text{R}}^{2}=1-{\sum_{i=1}^{n}\left(y_{i}-{\hat{y}}_{i}\right)}^{2}/{\sum_{i=1}^{n}\left(y_{i}-{\bar{y}}_{i}\right)}^{2}$$

(2) $$\text{RMSE}=\sqrt{{\sum_{i=1}^{n}\left(y_{i}-{\hat{y}}_{i}\right)}^{2}/n}$$

(3) $$\text{Bias}=\frac{1}{n}\sum_{i=1}^{n}\left(y_{i}-{\hat{y}}_{i}\right)$$

where ${\hat{y}}_{i}$ was the predicted values, $y_{i}$ was the measured values, and ${\bar{y}}_{i}$ was the mean of measured value. N was the sample number of validations.

Model building and validation were carried out by using the Scikit-learn library of Python 3.10. All graphs were obtained in OriginPro software 2019.

## 3 Results

### 3.1 Responds of photosynthetic traits of *Rhamnus leptophylla* to water stress

**Figure 2:**
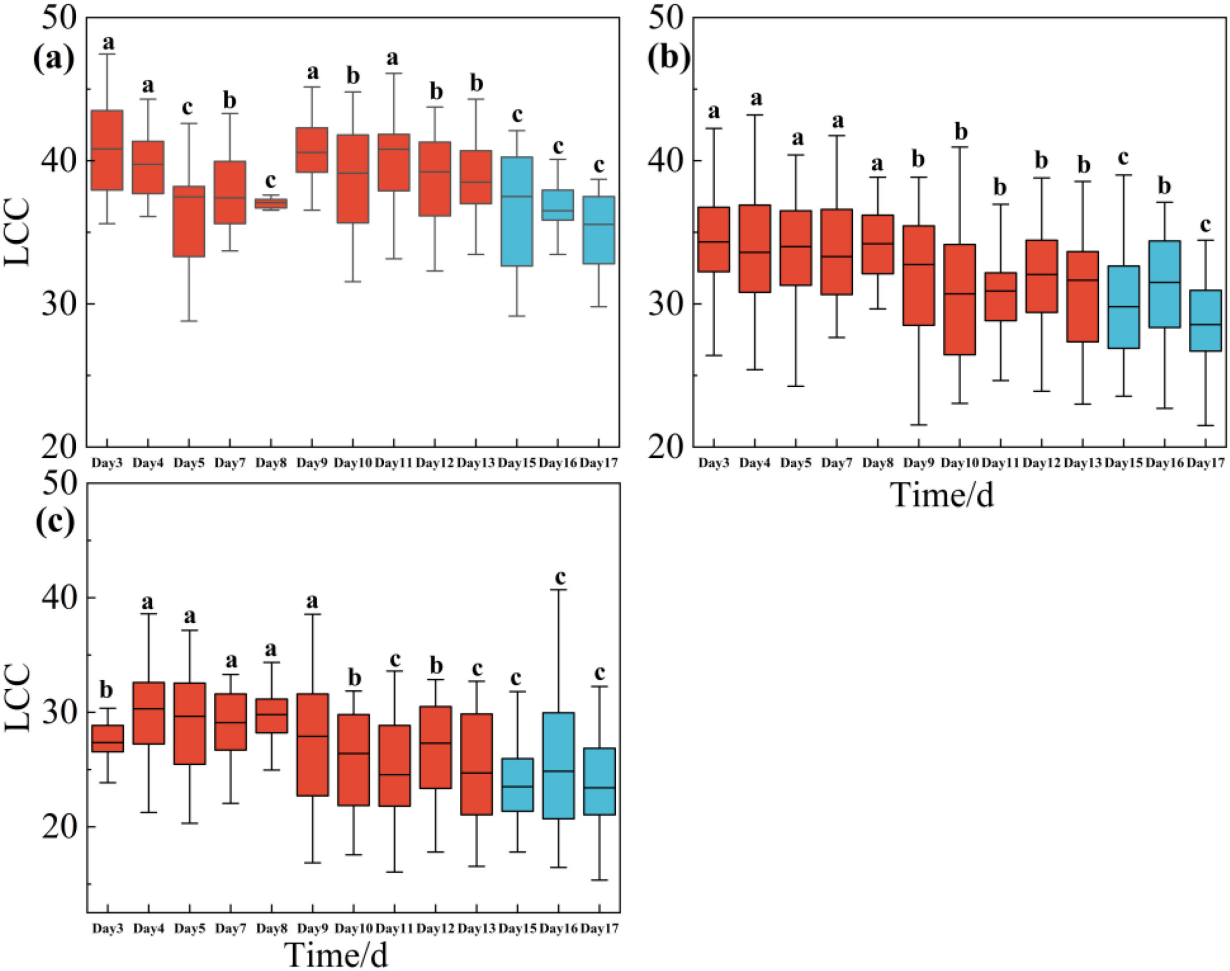
One-way ANOVA test results of LCC of *Rhamnus leptophylla* trees for the upper layer **(a)**, middle layer **(b)**, and lower layer **(c)** of different water stresses in the drought treatment period (i.e., Days 1, 3, 4 … until to day13) and rewatering period (i.e., Days 15, 16 and 17). The data are presented in the form of mean ± standard error, and significant differences are indicated by different letters.

**Figure 2** illustrated the effects of water stress to LCC across the upper layer, middle layer and lower layer during the drought treatment period (i.e., days 3, 4, 5,7, ……, until day 13) and rewatering period (i.e., days 15, 16 and 17). LCC decreased concurrently with the rapid decline in soil water content, regardless of whether the leaves were situated in the upper, middle, or lower layers. Leaves in the upper layers were more sensitive to drought, as LCC began to decline during the early stages of the drought (i.e., days 5). However, LCC begin to decrease significantly in day 9 and day 10 in middle layer and lower layer. After all the trees were re-watered in day 13, LCC continued to decrease obviously and did not recover as we expected in a short time (**Figure 2**).

**Figure 3:**
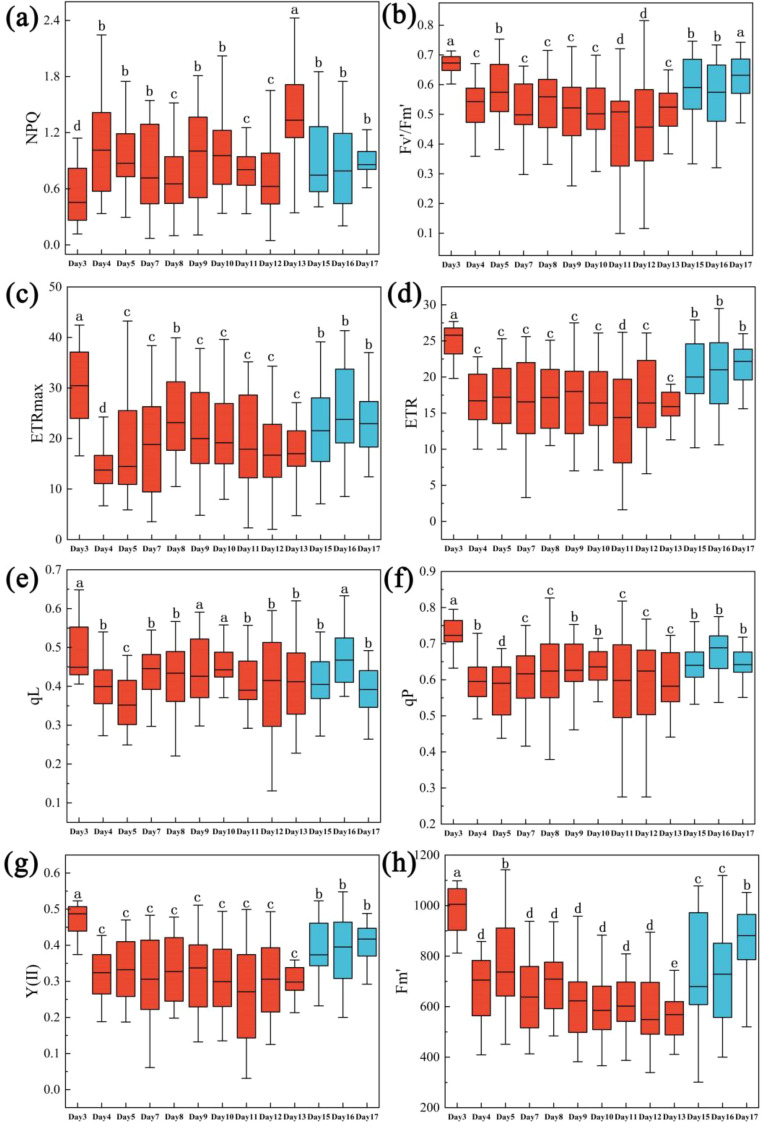
One-way ANOVA test results of ChlF of *Rhamnus leptophylla* trees for NPQ, Fv’/Fm’, and ETRmax of different water stresses in the drought treatment period (i.e., Days 1, 3, 4 … until to day13) and rewatering period (i.e., Days 15, 16 and 17). **(a–h)** were NPQ, Fv’/Fm’, ETRmax, ERT, qL, qP, Y(II) and Fm’. The data are presented in the form of mean ± standard error, and significant differences are indicated by different letters.

All the ChlF parameters decreased significantly and rapidly with reduced soil moisture content. NPQ initially increased and subsequently declined; Fv’/Fm’, ETRmax, ERT, qL, qP, Y(II) and Fm’ reduced significantly with severe drought. After trees were re-watered in day 13, these three ChlF parameters increased rapidly and almost returned to the initial level of early stage of drought from day 15 to day 17 (**Figures 3a–h**).

All the measured LCC and ChlF parameters had larger variation, which was benefit for building estimating model. The mean LCC value of upper layer, middle layer lower layer and average of all leaves was 38.43, 32.02, 26.70 and 31.16, respectively. The maximum LCC value was observed in upper layer with values of 48.75 and the minimum value was 15.35 of lower layer (**Table 2**). NPQ had the mean value of 0.91, the maximum value of 2.43 and the minimum value of 0.05. For Fv’/Fm’, the mean, maximum and minimum value was 0.54, 0.82 and 0.10, respectively. For ETRmax, the mean, maximum and minimum value was 20.37, 43.26 and 2.03, respectively (**Table 3**). And for ETR, qL, qP, Y(II) and Fm’, the mean value was 17.8, 0.42, 0.62, 0.34 and 680.93; the maximum value was 29.5, 0.65, 0.83, 0.55 and 1142.0; the minimum value was 1.60, 0.20, 0.28, 0.03 and 301.00, respectively (**Table 3**).

**Table 2:** Descriptive data of Leaf Chlorophyll Content.

Summary	Upper layer	Middle layer	Lower layer	All data
Mean	38.43	32.02	26.70	32.16
SD	3.79	3.75	4.69	6.21
Median	38.18	32.25	27.10	32.25
Maximum	48.75	40.40	38.60	47.45
Minimum	28.80	23.00	15.35	15.35
Coefficient Variation	0.10	0.12	0.18	0.19

**Table 3:** Descriptive data of Leaf Chlorophyll fluorescence parameters.

Summary	NPQ	Fv’/Fm’	ETR_max_	Fm’	qL	qP	Y(II)	ETR
Mean	0.91	0.54	20.37	680.93	0.42	0.62	0.34	17.80
SD	0.45	0.12	8.68	181.33	0.09	0.09	0.10	5.52
Median	0.85	0.54	19.24	660.00	0.42	0.63	0.34	18.10
Maximum	2.43	0.82	43.26	1142.00	0.65	0.83	0.55	29.50
Minimum	0.05	0.10	2.03	301.00	0.20	0.28	0.03	1.60
Coefficient Variation	0.50	0.23	0.43	0.27	0.20	0.15	0.31	0.31

### 3.2 Correlation between photosynthetic traits and raw hyperspectral reflectance, spectral prameters

**Figure 4:**
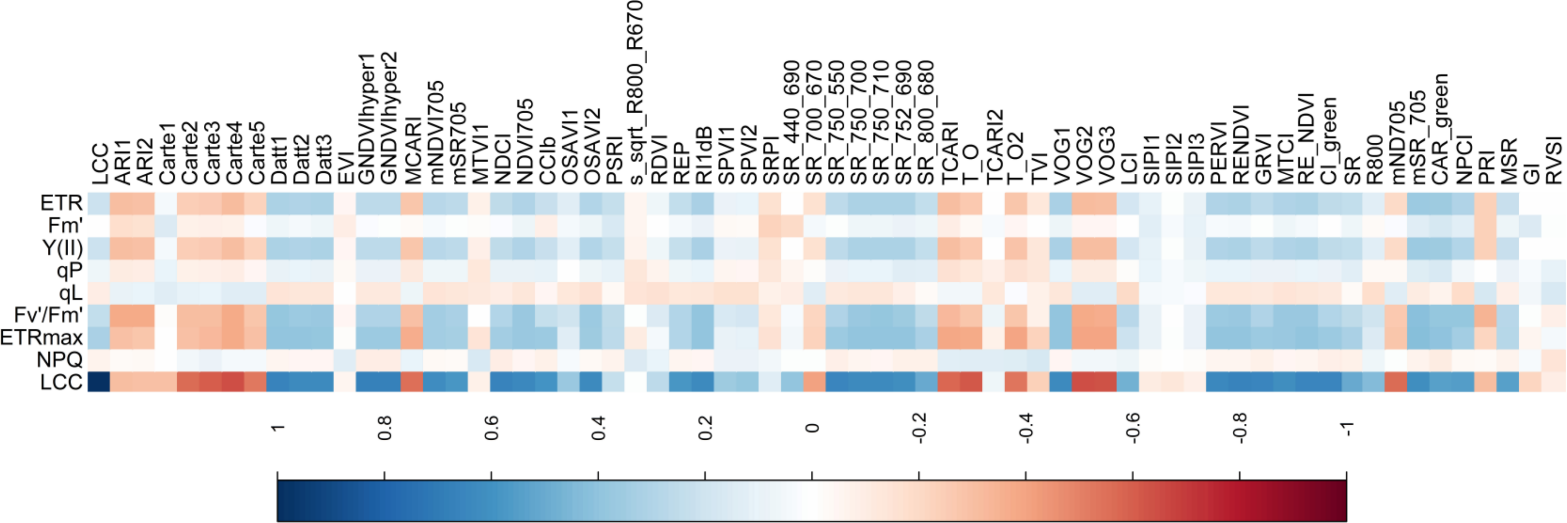
Correlation coefficients between LCC, NPQ, Fv’/Fm’, ETRmax, Fm’, qL, qP, Y(II), ERT and hyperspectral parameters.

In general, LCC and raw hyperspectral reflectance showed high negative correlation coefficients (r) in the visible spectrum (approximately 500 nm to 710 nm) and low correlation in the infrared region (approximately 760 nm to 1000 nm). The r value increased and reached a maximum in 500-600nm, decreased sharply in 680nm and then continue to increase in 700nm (**Figure 4**). Different datasets from various canopy layers had a significant impact on the correlation between LCC and raw reflectance. |r| with all data was the highest and the maximum was 0.57 in 573nm. The second was the middle layer and the maximum value was 0.38 in 569nm. Upper layer had the lowest correlation with the highest |r| value of 0.19 (**Supplementary Figure S1**).

**Figure 5:**
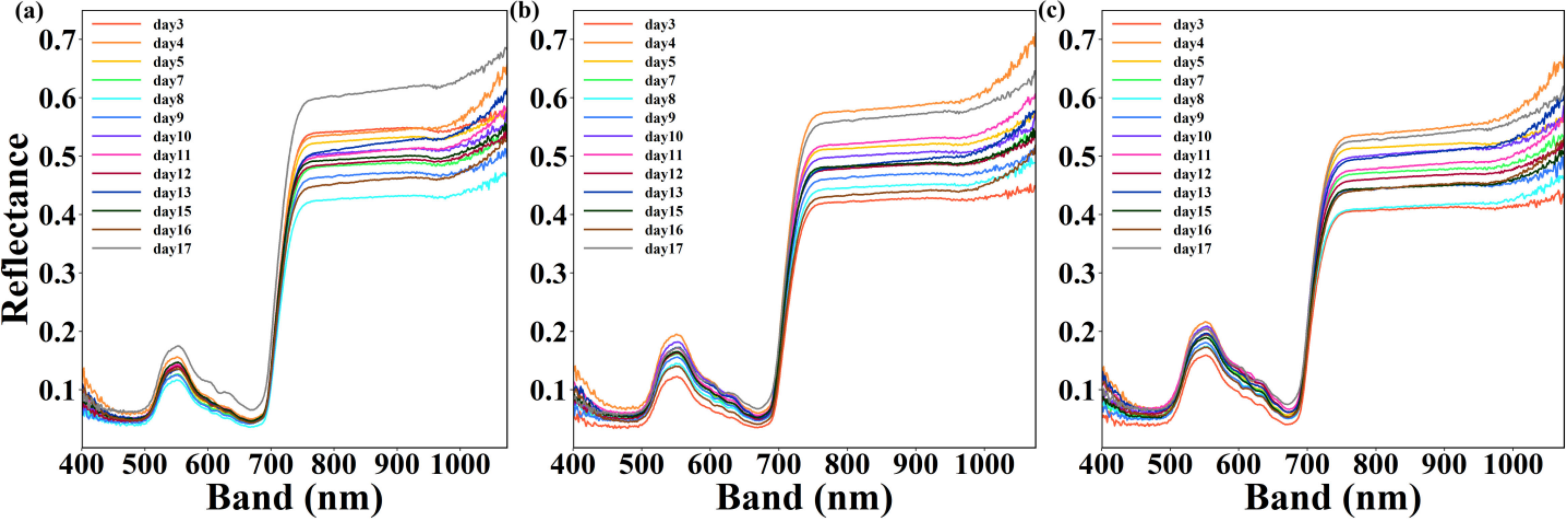
Comparison between the mean spectral reflectance and absorptance at 400–1100 nm for each of different water stress from day 3 to day 17. **(a–c)** represent upper layer, middle layer and lower layer.

Similarly, ChlF parameters exhibited a negative correlation with hyperspectral reflectance within the visible spectrum. Specifically, for NPQ, the highest absolute correlation coefficient (|r| > 0.17) was observed in the wavelength range of 400–550 nm (**Supplementary Figure S2a**). For Fv’/Fm’, |r| values exceeded 0.25 in the range of 574–636 nm, with a distinct peak at 617 nm (r = - 0.29) (**Figure 5b**). For ETRmax, |r| values greater than 0.3 were detected in the range of 492–634 nm, accompanied by a peak in the range of 700–709 nm (|r| > 0.35) (**Supplementary Figure S2c**). For Y(II) and ETR, |r| values exceeded 0.2 in the range of 330–638 nm, with a similar peak observed in the range of 694–714 nm, consistent with the trend observed for ETRmax (**Supplementary Figures S2g, h**). In contrast, qL and qP showed relatively low correlations with raw hyperspectral reflectance, with |r| values exceeding 0.1 only in the ranges of 736–1075 nm and 325–506 nm, respectively (**Supplementary Figures S2e, f**).

We also analyzed the correlation between LCC, NPQ, Fv’/Fm’, ETRmax, Fm’, qL, qP, Y(II), ERT and hyperspectral parameters, which were presented in **Figure 4**. LCC had the strongest positive correlation with GNDVI and GNDVIhyper1 and the highest r value of 0.6785; mSR_705 was the most relevant to ERT, Y(II), Fv’/Fm’ and ETRmax the highest r value of 0.3652, 0.3623, 0.4138 and 0.4289, respectively. qL and qP had lower relation with hyperspectral parameters and the highest r value was 0.1629 and -0.1919 in CAR_green and LCI. Considered the correaltion between all photosynthetic traits and hyperspectral parameters, we selected the top 22 hyperspectral parameters which were mSR_705, CAR_green, SR(735/720), VOG1, Datt1, SR(750/710), RENDVI, RE_NDVI, Carte4, SR(750/700), SR(752/690), Datt3, NDVI705, VOG2, PERVI, Datt2, VOG3, MTCI, TCARI, NPCI, OSAVI2 and GNDVIhyper1. And we tracked the drought by using these 22 hyperspectral parameters.

### 3.3 Variation in leaf reflectance spectra for drought and tracking of leaf hyperspectral reflectance to drought

Water stress caused continuous and dynamic changes of the mean spectral reflectance and absorptance over time. From day 3 to day 17, two band range including 540-560nm and 750-1100nm were found to distinguish the different water stress whatever the leaves were in upper layer, middle layer or lower layer (**Figures 5a–c**).

Most vegetation indices decreased with drought and increased after re-watering expect for VOG2, VOG3, and NPCI (**Supplementary Table S1**). After suffering from a 4-day drought, mSR_705, CAR_green, Datt1, Datt2, Datt3, SR(750/700), NPCI, and GNDVIhyper1 present decreased significantly comparing to day 3. SR(752/690) was the most sensitive to drought and present obvious decrease in day 5. VOG1, MTCI and OSAVI2 began to decrease significantly in day 8. SR(735/720) and NDVI705 decreased in day 10. In day 12, almost all the vegetation index reached the minimum value. But SR(752/690) was in day 9 and mSR_705, CAR_green, Datt1, Datt2, Datt3, SR(750/700), NPCI, and GNDVIhyper1 was in day 10 (**Supplementary Table S1**).

### 3.4 Parameter selection

**Figure 6:**
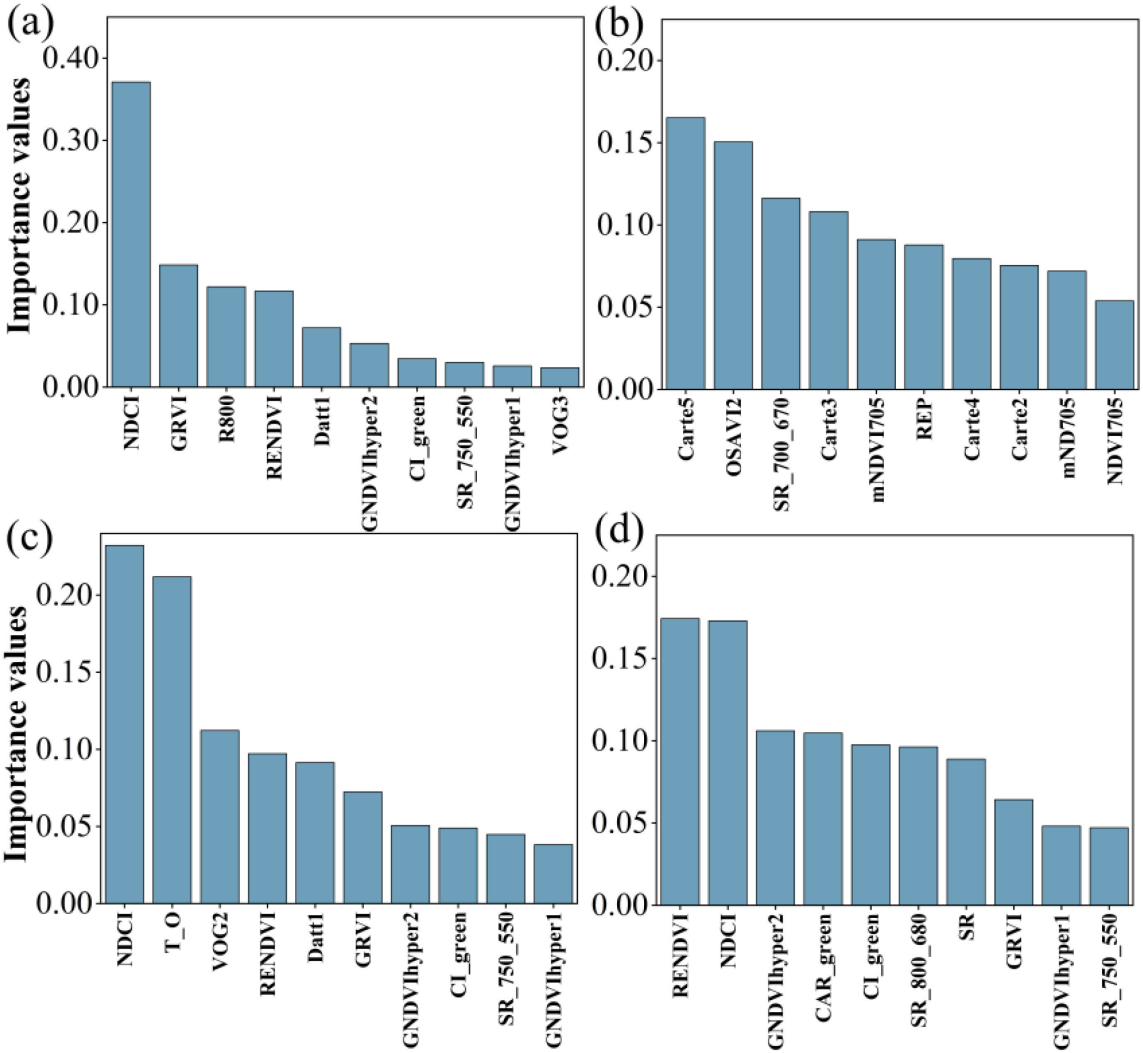
Ranking the importance of 10 sensitive hyperspectral parameters. **(a-d)** indicated all LCC, LCC of upper layer, LCC of middle layer and LCC of lower layer.

**Figure 6** showed that ten sensitive hyperspectral parameters for LCC estimation were ranked by importance to identify the top two parameters for different layers. Those were NDCI and GRVI for all data, Carte5 and OSAVI2 for upper layer, RENDVI and VOG2 for middle layer, CAR_green and SR (800, 680) for lower layer, respectively. These hyperspectral parameters were selected for estimating LCC.

**Figure 7:**
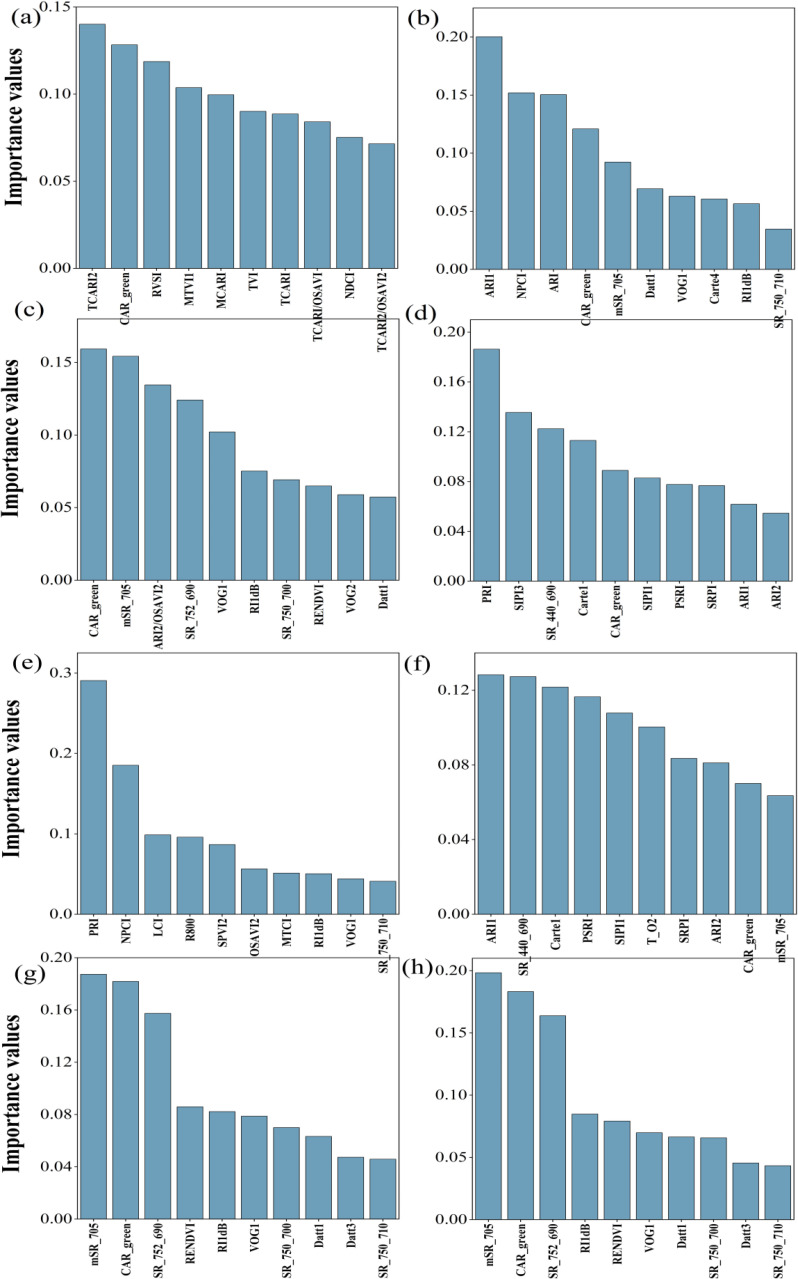
Ranking the importance of 10 sensitive hyperspectral parameters. **(a–h)** were NPQ, Fv’/Fm’, ETRmax, Fm’, qL, qP, Y(II) and ETR respectively.

**Figure 7** indicated the ten sensitive hyperspectral parameters of ChlF estimation were ranked according to importance. The top two parameters for NPQ, Fv’/Fm’, ETRmax, Fm’, qL, qP, Y(II) and ETR were TCARI2 and CAR_green, ARI1 and NPCI, CAR_green and mSR_705, PRI and SIPI3, PRI and NCPI, ARI1 and SR(440/690), mSR_705 and CAR_green, mSR_705 and CAR_green, respectively.

### 3.5 Multivariate linear regression for estimating LCC of *Rhamnus leptophylla*

According to the importance ranking in **Figure 6**, the top five sensitive parameters were selected for MLR models. The R^2^ and RMSE were 0.505 and 21.95 for all data, 0.013 and 12.78 for upper layer, 0.126 and 12.10 for middle layer, 0.00 and 17.89 for lower layer. And the highest R^2^ was only 0.505 (**Table 4**).

**Table 4:** Performance of MLR for estimating LCC by using different data.

	Models	R^2^	RMSE
All data	y=0.816 + 26.433×*x_1_* +1.670×*x_2_* +7.184×*x_3_*-18.214×*x_4_* +20.324×*x_5_*	0.505	21.95
Upper layer	y=68.684-20.275×*x_1_*-25.029×*x_2_* +7.185×*x_3_*-17.043×*x_4_*-0.513×*x_5_*	0.013	12.78
Middle layer	y=38.062-9.402×*x_1_* +3.945×*x_2_*-8.231×*x_3_*-2.687×*x_4_*-49.215×*x_5_*	0.126	12.10
Lower layer	y=20.306-0.686×*x_1_* +3.776×*x_2_* +52.041×*x_3_*-0.372×*x_4_*-41.383×*x_5_*	0.000	17.89

*x_1_*, *x_2_*, and *x_3_* represented the parameters of the best-fitting model. For all data, *x_1_*, *x_2_*, *x_3_, x_4_* and *x_5_* were NDCI, GRVI, R800, RENDVI, Datt1; For upper layer, *x_1_*, *x_2_*, *x_3_, x_4_* and *x_5_* were Carte5, OSAVI2, SR(700, 670), Carte3, mNDVI705; For middle layer, *x_1_*, *x_2_*, *x_3_, x_4_* and *x_5_* were NDVI, CI_green, TCARI/OSAVI, SR(750, 550), VOG2; For lower layer, *x_1_*, *x_2_*, *x_3_, x_4_* and *x_5_* were SR(800, 680), RENDVI, DCI, CAR_green, GNDVIhyper2.

Overall, the MLR presented to be very weak in estimating ChlF parameters of *Rhamnus leptophylla* (**Table 5**). The R^2^ of NPQ, Fv’/Fm’, ETRmax, Fm’, qL, qP, Y(II) and ETR was 0.055, 0.208, 0.065, 0.134, 0.050, 0.008, 0.142, and 0.133 respectively.

**Table 5:** Performance of MLR for estimating Chlorophyll fluorescence parameters.

	models	R^2^	RMSE
NPQ	y=0.938-0.091×*x_1_*-0.091×*x_2_* +4.388×*x_3_* +2.664×*x_4_*-0.930×*x_5_*	0.055	0.217
Fv’/Fm’	y=0.530-0.022×*x_1_* +0.353×*x_2_*-0.022×*x_3_* +0.018×*x_4_*-0.003×*x_5_*	0.208	0.011
ETR_max_	y=6.342 + 12.718×*x_1_* +2.293×*x_2_*-4.172×*x_3_* +3.285×*x_4_*-2.139×*x_5_*	0.065	77.83
Fm’	y=-331.799-3875.452×*x_1_*-241.854×*x_2_*-0.555×*x_3_* + 3.932×*x_4+_* 1069.851×*x_5_*	0.134	33250.991
qL	y=0.419 + 0.203×*x_1_*-0.291×*x_2_*-0.006×*x_3_* +2.079×*x_4_*-1.401×*x_5_*	0.050	0.006
qP	y=-0.160-0.008×*x_1_* + 0.054×*x_2+_* 0.655×*x_3_*-0.054×*x_4+_* 0.038×*x_5_*	0.008	0.008
Y(II)	y=-0.431 + 0.026×*x_1_*-0.725×*x_2_*-0.028×*x_3_* +0.137×*x_4+_* 0.630×*x_5_*	0.142	0.01
ETR	y=-57.013-2.17×*x_1+_* 2.149×*x_2_*-1.515×*x_3_* +72.218×*x_4_*-33.321×*x_5_*	0.133	26.936

*x_1_*, *x_2_*, and *x_3_* represented the parameters of the best-fitting model. For NPQ, *x_1_*, *x_2_*, *x_3_, x_4_* and *x_5_* were TCARI2,CAR_green,RVSI, MTVI1,MCARI; For Fv’/Fm’, *x_1_*, *x_2_*, *x_3_, x_4_* and *x_5_* were ARI1, NPCI, ARI, CAR_green, mSR_705; For Fm’, x1, x2, x3, x4 and x5 were PRI, SR(440/690), Carte1, CAR_green and SIPI3; For qL, x1, x2, x3, x4 and x5 were PRI, NPCI, LCI, R800, SPVI2; For qP, x1, x2, x3, x4 and x5 were PSRI, SR(440/690), ARI1, Carte1; For Y(II), x1, x2, x3, x4 and x5 were CAR_green, RENDVI, SR(752/690), mSR_705, RVI; For ETR, x1, x2, x3, x4 and x5 were mSR705, CAR_green, SR(752/690), RVI, RENDVI.

### 3.6 Machine-learning algorithms for predicting leaf chlorophyll content

A comparison of the three machine learning algorithms revealed that RF algorithm exhibited the best regression performance, characterized by the highest R² and the lowest RMSE, rRMSE and Bias. For LCC, the R^2^ value of RF was 0.895 for all samples, 0.697 for upper layer, 0.902 for middle layer and 0.795 for lower layer (**Table 6**); and the KNN were 0.608 for all leaves, 0.110 for upper layer, 0.243 for middle layer, 0.00 for lower layer; and the SVR were 0.615 for all leaves, 0.267 for upper layer, 0.145 for lower layer, and 0.00 for lower layer, respectively (**Table 6**). RF not only obtained highest R^2^ but also lowest RMSE, r RMSE and Bias whatever the data was acquired of all leaves, upper layer, middle layer or lower layer. KNN and SVM presented to be weaker in estimating LCC comparing to RF.

**Table 6:** Performance of different machine-learning algorithms employed for the LCC estimation.

		RF	KNN	SVM	Parameters
All leaves	R^2^	0.895	0.608	0.615	NDCI, GRVI, R_800_, RENDVI, Datt1
	RMSE	2.155	4.012	4.131	
	rRMSE	0.066	0.124	0.130	
	Bias	-0.057	0.034	-0.028	
Upper layer	R^2^	0.697	0.110	0.267	Carte5, OSAVI2, SR(700, 670), Carte3, mNDVI705
	RMSE	1.982	3.361	3.079	
	rRMSE	0.051	0.087	0.080	
	Bias	-0.016	0.210	-0.400	
Middle layer	R^2^	0.902	0.243	0.145	NDCI, RENDVI, TCARI/OSAVI, Datt1, VOG2
	RMSE	1.213	3.295	3.441	
	rRMSE	0.036	0.103	0.107	
	Bias	0.081	0.094	0.152	
Lower layer	R^2^	0.795	0.00	0.00	SR(800, 680), RENDVI, DCI, CAR_green, GNDVIhyper2
	RMSE	1.859	4.218	4.139	
	rRMSE	0.069	0.156	0.153	
	Bias	0.598	-0.234	0.138	

**Figure 8:**
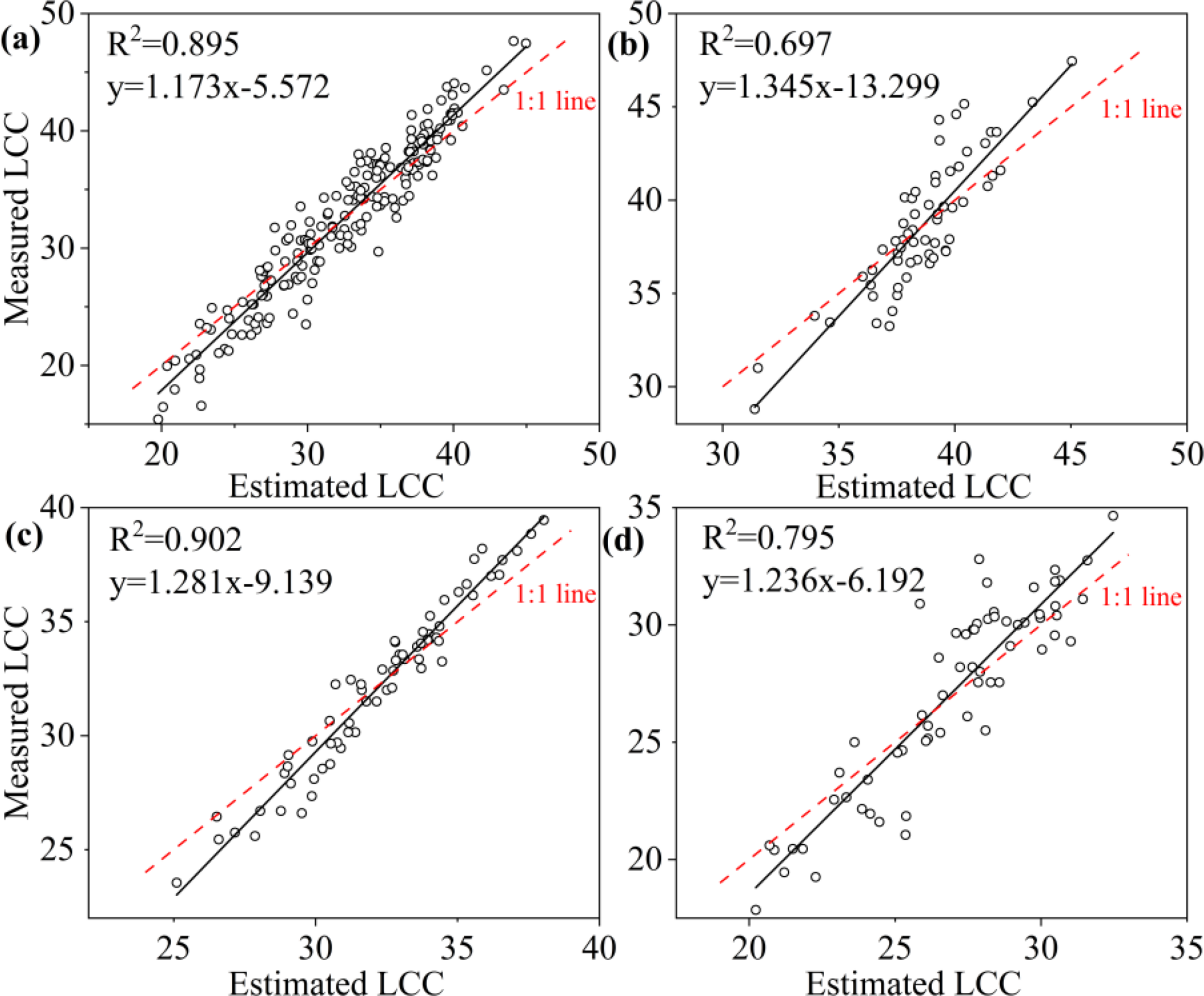
Estimated and measured along the 1:1 line of the RF model. **(a)**, **(b)**, **(c)**, and **(d)** were all samples, upper layer, middle layer and lower layer, respectively.

**Figure 9:**
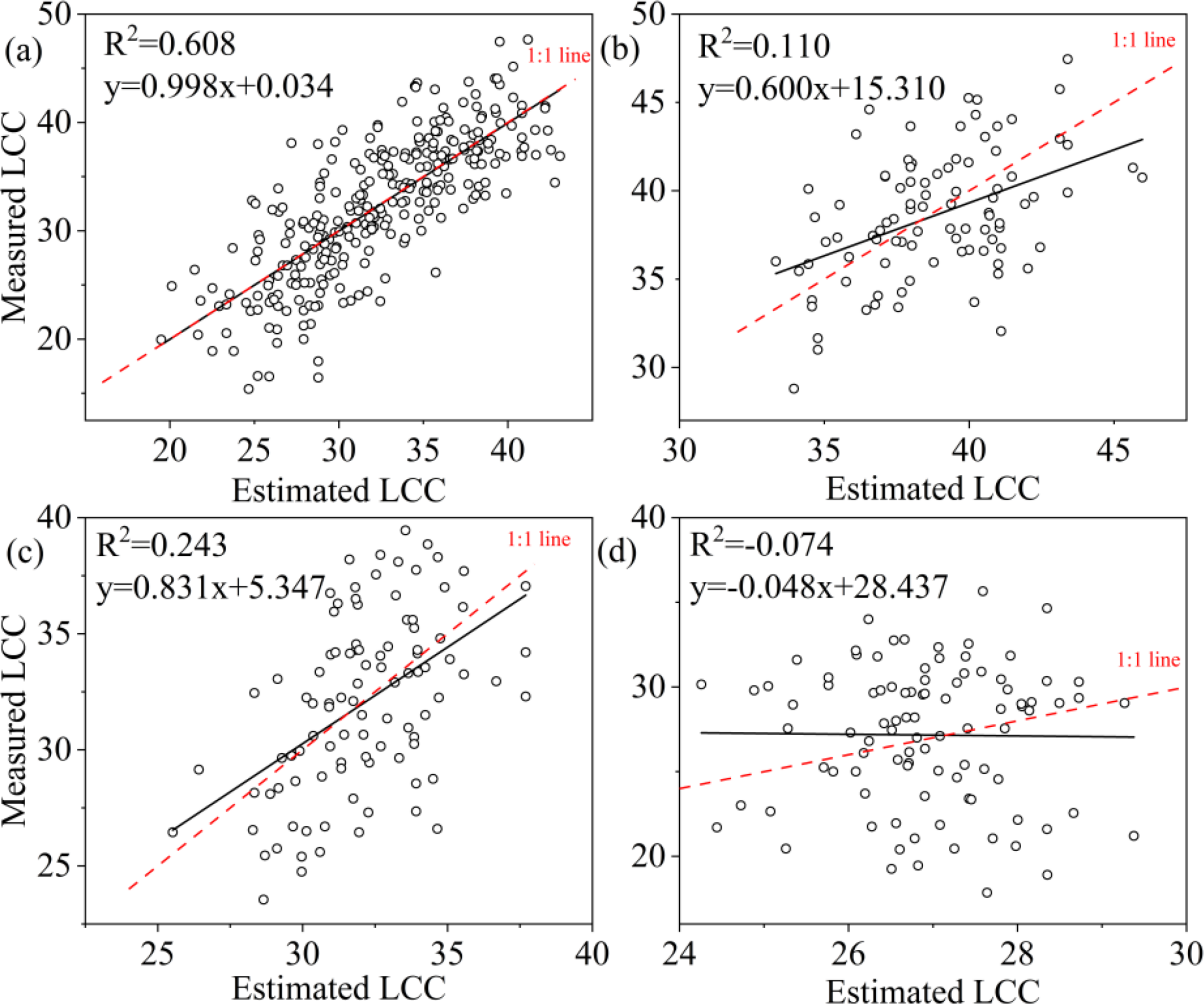
Estimated and measured along the 1:1 line of the KNN model. **(a)**, **(b)**, **(c)**, and **(d)** were all samples, upper layer, middle layer and lower layer, respectively.

**Figure 10:**
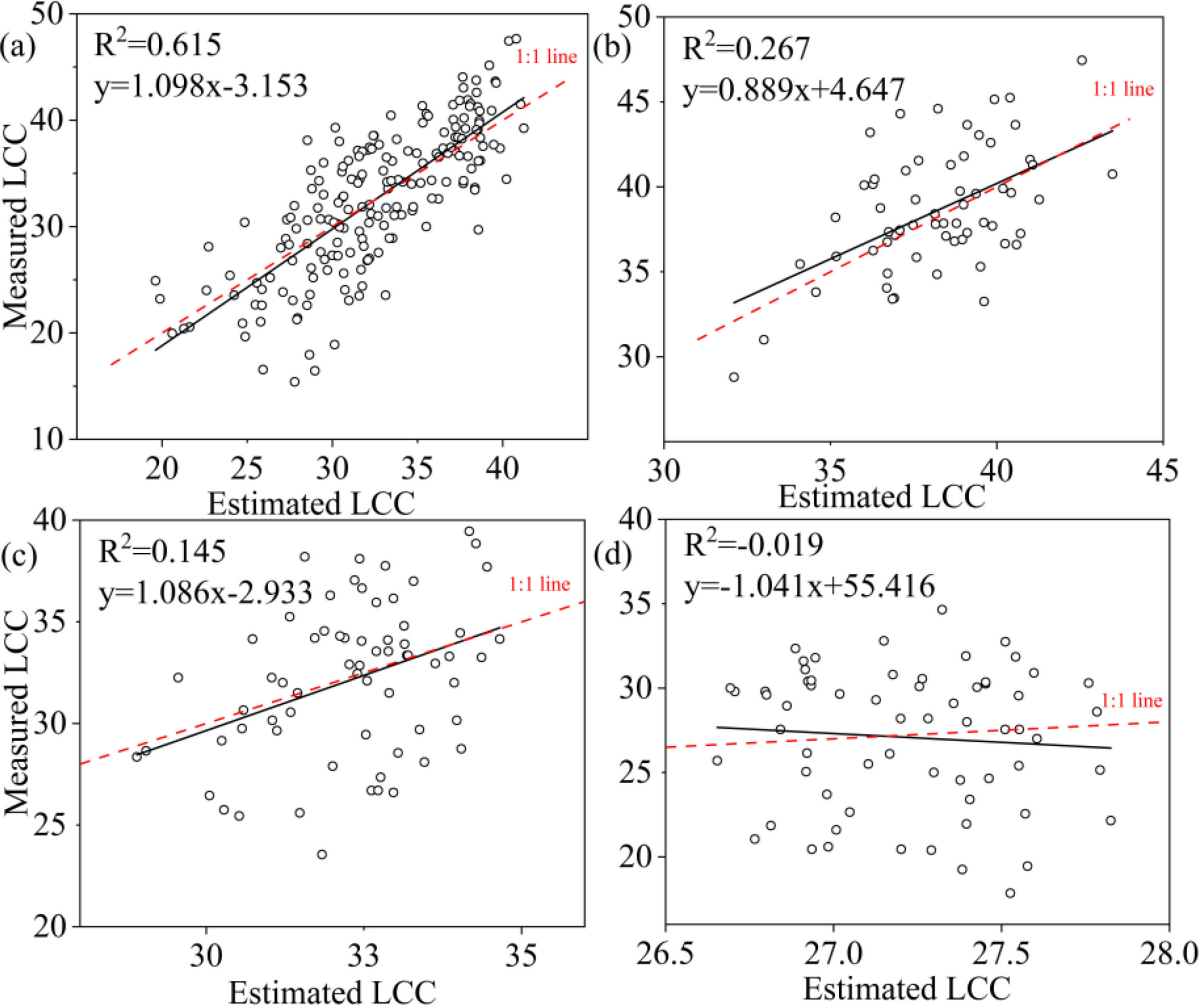
Estimated and measured along the 1:1 line of the SVM model. **(a)**, **(b)**, **(c)** and **(d)** were all samples, upper layer, middle layer and lower layer, respectively.

To ascertain the relationship between observed and predicted LCC, the regression values were plotted (**Figures 8**–**10**). For all leaves, RF, KNN and SVM presented similar trends to a 1:1 relationship (**Figures 8a**, **9a**, **10a**). For upper, middle and lower layer, KNN and SVM did not show a similarity to a 1:1 relationship (dashed-line—**Figures 8b–d**, **Figures 9b–d**, **Figures 10b–d**). Predictions of RF were comparatively well related to the observed LCC for all leaves, upper layer, middle layer and lower layer.

### 3.7 Machine-learning algorithms for predicting chlorophyll fluorescence parameters

**Figure 11:**
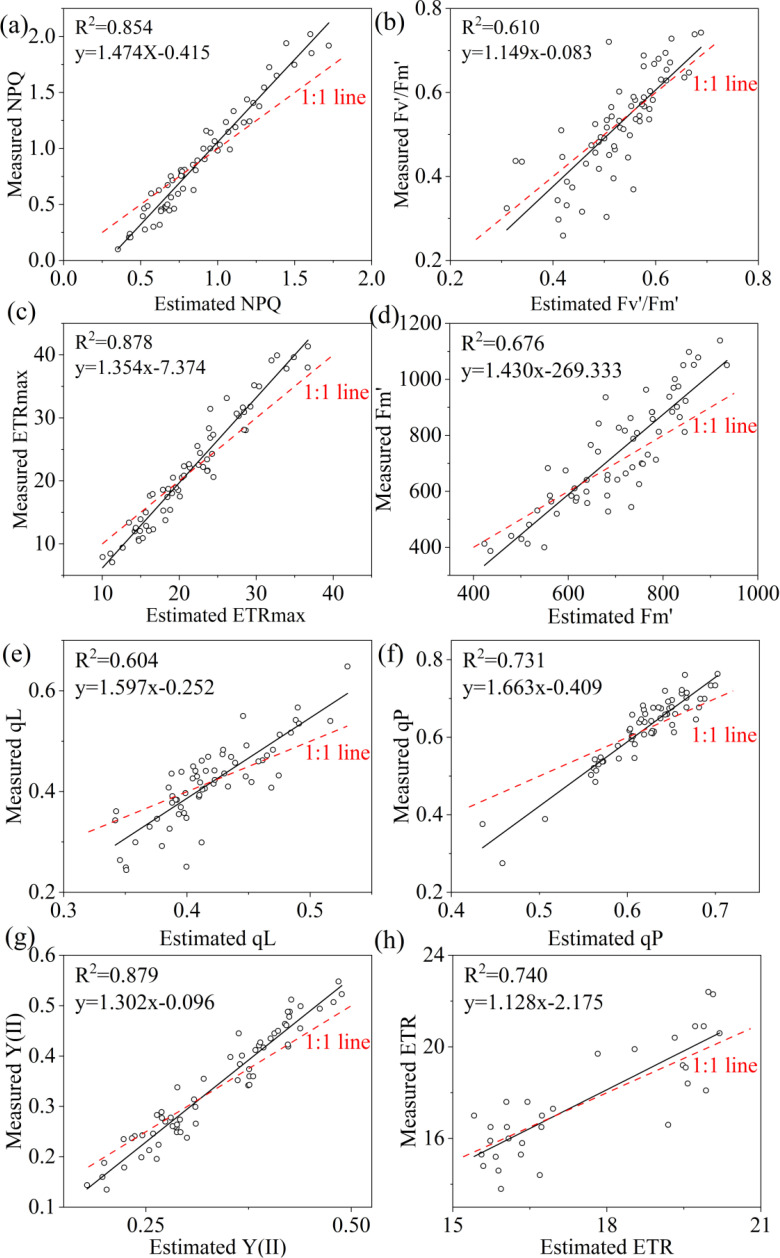
Estimated and measured along the 1:1 line of the RF model. **(a–h)** were NPQ, Fv’/Fm’, ETRmax, Fm’, qL, qP, Y(II) and ETR respectively.

Three machine-learning algorithms were also used for estimating the ChlF parameters including NPQ, Fv’/Fm’, ETRmax, qL, qP, Y(II) and ETR. Similarly, RF performed to be the best, achieving the highest R^2^ of 0.854, 0.610, 0.878, 0.676, 0.604, 0.731,0.879,0.740 for NPQ, Fv’/Fm’, ETRmax, Fm’, qL, qP, Y(II) and ETR, respectively. Additionally, it exhibited the lowest RMSE, rRMSE, and bias when compared to other algorithms (**Table 7**). R^2^ of KNN were 0.011 for NPQ, 0.202 for Fv’/Fm’, 0.112 for ETRmax, 0.178 for Fm’, 0.156 for qL, 0.118 for qP, 0.204 for Y(II), 0.072 for ERT; and the R^2^ of SVM were 0.057 for NPQ, 0.199 for Fv’/Fm’, 0.022 for ETRmax, 0.240 for Fm’, 0.195 for qL, 0.233 for qP, 0.109 for Y(II), 0.210 for ERT, respectively. The regression analysis based on RF algorithm between the observed and predicted ChlF parameters were plotted in **Figure 11**.

**Table 7:** Performance of different machine-learning algorithms employed for the estimation of Chlorophyll fluorescence parameters.

		RF	KNN	SVM	Parameters
NPQ	R^2^	0.854	0.011	0.057	TCARI2,CAR_green,RVSI, MTVI1,MCARI
	RMSE	0.183	0.461	0.466	
	rRMSE	0.206	0.511	0.053	
	Bias	0.007	-0.034	-0.024	
Fv’/Fm’	R^2^	0.610	0.202	0.199	ARI1,NPCI,ARI,CAR_green, mSR_705
	RMSE	0.074	0.103	0.106	
	rRMSE	0.141	0.192	0.202	
	Bias	0.006	0.001	0.014	
ETR_max_	R^2^	0.878	0.112	0.022	mSR_705, CAR_green, TCARI/OSAVI, VOG1,SR(752/690)
	RMSE	3.185	8.784	9.021	
	rRMSE	0.147	0.418	0.416	
	Bias	-0.143	-1.099	-2.075	
Fm’	R^2^	0.676	0.178	0.240	PRI, SR(440/690), Carte1, CAR_green, SIPI3
	RMSE	111.613	183.850	188.851	
	rRMSE	0.152	0.264	0.257	
	Bias	-10.657	-17.870	-88.947	
qL	R^2^	0.604	0.156	0.195	PRI, NPCI, LCI, R800, SPVI2
	RMSE	0.050	0.078	0.082	
	rRMSE	0.121	0.186	0.198	
	Bias	0.001	0.006	0.002	
qP	R^2^	0.731	0.118	0.233	PSRI, SR(440/690), ARI1, Carte1
	RMSE	0.471	0.101	0.092	
	rRMSE	0.076	0.165	0.150	
	Bias	-0.001	0.015	0.008	
Y(II)	R^2^	0.879	0.204	0.109	CAR_green, RENDVI, SR(752/690), mSR_705, RVI
	RMSE	0.038	0.108	0.103	
	rRMSE	0.112	0.324	0.303	
	Bias	-0.002	-0.002	-0.011	
ETR	R^2^	0.740	0.072	0.210	mSR705, CAR_green, SR(752/690), RVI, RENDVI
	RMSE	1.307	5.337	4.956	
	rRMSE	0.074	0.303	0.286	
	Bias	-0.017	0.202	0.265	

## 4 Discussion

Climate change caused by global warming reduced soil moisture and resulting aggravated droughts (Trenberth et al., 2014; Samaniego et al., 2018). Drought resistance is a combination of physiological and biochemical adaptations that can be reflected in the plants’ spectral signature (**Figure 4** and **Supplementary Table S1**) (Zovko et al., 2019). It is important for mitigating the damage of drought by monitoring the effect of drought to plant with non-destructive and rapid way before symptoms can be seen with eye. Photosynthetic response to drought and its sensitivity to soil water deficit (**Figures 2**, **3**) (Zhou et al., 2021). The spectral properties of plants, as a visual reflection of the chemical and physiological state of leaves, show high sensitivity to environmental changes (Zovko et al., 2019; Zhang et al., 2024). So, this study combined photosynthetic factor including LCC, ChlF and leaf hyperspectral reflectance of *Rhamnus leptophylla* which experienced 13 days drought and 3 days re-watering for attempting to track the physiological changes with hyperspectral reflectance and make models to monitoring these variations. *Rhamnus leptophylla* is a common shrub or small tree in the Three Gorges area and its assessment for monitoring of drought resistance decided due to its potential for side slope protection in fluctuation zone. It is urgent to quickly and accurately monitor the response of *Rhamnus leptophylla* to drought, as its ability to withstand drought and the mechanisms involved remain unknown.

Drought significantly can impact chlorophyll content and chlorophyll fluorescence in plants, leading to alterations in photosynthetic efficiency. Our results showed that LCC, Fv’/Fm’, ETRmax, Fm’, qL, qP, Y(II) and ETR decreased with decreasing soil moisture content; NPQ increased firstly and then decreased as severe drought (**Figures 2**, **3**). Because under drought conditions, reduced chlorophyll synthesis and closed stomata were contributed to lower chlorophyll concentrations. Fv’/Fm’ represents the maximum photochemical efficiency of PSII under light adaptation, which can reflect the photosynthetic capacity of plants under light conditions., FV’/FM’ tended to decrease under drought stress (**Figure 3b**). Drought stress can bring stomatal closure of plant leaves contributing to the reduction of intercellular CO_2_ concentration, which makes the fixation of CO_2_ in the dark reaction inhibited. Light response and electron transport chain of photosynthesis was also affected. This blocked state decreased the opening ratio of the PSII reaction center, which will decrease the value of Fv’/Fm’. ETRmax showed a decreasing trend with the aggravation of drought (**Figure 3c**). This was because that drought can damage electron transport chain in the photosynthetic system. Salvucci and Crafts-Brandner (2004) showed that drought stress significantly reduces the photosynthetic capacity of plants, including ETRmax.

There was a high negative correlation between the raw hyperspectral reflectance (green and red bands) and LCC in this study, which may be attributed to several physiological and spectral mechanisms inherent to plant leaves. These mechanisms are influenced by leaf structural characteristics and external environmental factors such as moisture conditions and soil nutrient (Gitelson and Merzlyak, 1997; Sims and Gamon, 2002; Ustin and Gamon, 2010). Leaf internal structure and water content significantly influence spectral reflectance. For instance, increased leaf thickness or lower water content may lead to an apparent negative correlation between hyperspectral bands and chlorophyll levels. Drought stress can modify the traditional correlation between chlorophyll content and hyperspectral reflectance. Changes in leaf structure and water content induced by drought can cause an overall shift in spectral characteristics, thereby leading to the negative relationship between LCC and reflectance (Sims and Gamon, 2002; Li et al., 2024b). The canopy layer exerts a remarkable influence on the LCC and its corresponding estimating accuracy. This conclusion is applicable to both seedlings and mature trees, regardless of the growth stage (Zhou et al., 2021; Li et al., 2023). These results may be contributed to leaves of different maturity level at distinct canopy levels. When leaves are confronted with drought stress, the responses of leaves with varying maturity levels to drought, which in turn results in diverse changes in chlorophyll content (**Table 2**). Leaves at different levels respond differently to drought stress in terms of their spectral characteristics (**Figure 5**). NPQ was related with spectral reflectance in blue band (400-550nm), Fv’/Fm’ was in green and red band(570-635nm) and ETRmax was in blue, green and red edge band (480-630nm, 690-715nm), respectively. That was because the chlorophyll absorption and reflection characteristics of light in this region are remarkable in visible light region. These results were similar to other studies which also revealed the utility of the visible light region for estimating photosynthetic characteristic. The green (500–599 nm) and red (601–696 nm) regions were selected for ФF and qL, respectively (Song et al., 2024). 486 nm, 668 nm, 690 nm and725 nm were used for constructing new index for estimating Chlorophyll Fluorescence Parameters (Zheng et al., 2021). However, selection of hyperspectral band or hyperspectral parameters was uncertain and specific band for LCC or ChlF parameters was necessary to determine.

In this study, we established MLR and three machine learning models (RF, KNN and SVM) using field-measured hyperspectral data to estimate LCC and eight ChlF parameters. Our result indicated that RF performed to be the best in estimating LCC and ChlF (**Tables 6**, **7**). R^2^ of 0.895, 0.854, 0.610, 0.878, 0.676, 0.604, 0.731,0.879,0.740 for LCC, NPQ, Fv’/Fm’, ETRmax, Fm’, qL, qP, Y(II) and ETR, respectively (**Figure 11**). RF is composed of multiple trees trained through bagging and a random variable selection process. RF was proved to be robustness against outliers and noise (Maimaitijiang et al., 2020; Khruschev et al., 2022). Moreover, it is excellent in handle the substantial common multivariate collinearity inherent in the functional relationship between spectral variables and biophysical or biochemical parameters (Liang et al., 2016). RF as a supervised learning technique for regression has been already consistently reported to obtain high accuracy in estimating photosynthetic parameters (An et al., 2020; Zhou et al., 2021; Li et al., 2023; Shi et al., 2023). Canopy layer influenced the LCC and corresponding estimation accuracy and need to be considered seriously. This conclusion was indicated in photosynthesis and nutrients utility of citrus trees (Zhou et al., 2021; Dian et al., 2023; Li et al., 2024a).

## 5 Conclusions

Linking leaf hyperspectral reflectance and plant photosynthetic traits can achieve accurate and non-destructive drought monitoring before visible symptoms appeared in plants. In this study, photosynthetic traits including LCC, NPQ, Fv’/Fm’, ETRmax, Fm’, qL, qP, Y(II) and ETR presented rapid decrease with reduced soil moisture. Chlorophyll fluorescence was more sensitive than LCC. The original reflectance and hyperspectral SVIs had high correlation with LCC and chlorophyll fluorescence parameters. Spectral bands in 540-560nm and 750-1100nm can distinguish different water stress. Selected hyperspectral SVIs including mSR_705, CAR_green, Datt1, Datt2, Datt3, SR(750/700), NPCI, and NDVI present can effectively track the water stress after plant experienced 6 days’ drought. SR(752/690) was the most sensitive to drought and present obvious decrease in day 4. Photosynthetic traits such as LCC, NPQ, Fv’/Fm’, ETRmax, Fm’, qL, qP, Y(II) and ETR could be estimated with the highest precision by applying hyperspectral leaf reflectance and RF models compared to MLR, SVM and KNN. And the canopy layer should be considered when physiological factors were estimated. In short, hyperspectral reflectance was very effective in testing drought advanced by combining physiological traits. To our knowledge, this is one of the first applications of hyperspectral parameters as indicators for drought and input for the estimation of photosynthetic traits in *Rhamnus leptophylla* and provides a basis for expanding the applications to other observing platforms, such as unmanned aerial and satellite remote sensing.

## Data Availability

The original contributions presented in the study are included in the article/supplementary material. Further inquiries can be directed to the corresponding author.
