# Supplementary material for: Combine photosynthetic characteristics and leaf hyperspectral reflectance for early detection of water stress
Source: Front Plant Sci. 2025 Apr 9;16:1520304. doi: 10.3389/fpls.2025.1520304 (PMC12014561; doi:10.3389/fpls.2025.1520304)
Supplement: Supplementary file 1 [file DataSheet1.docx]

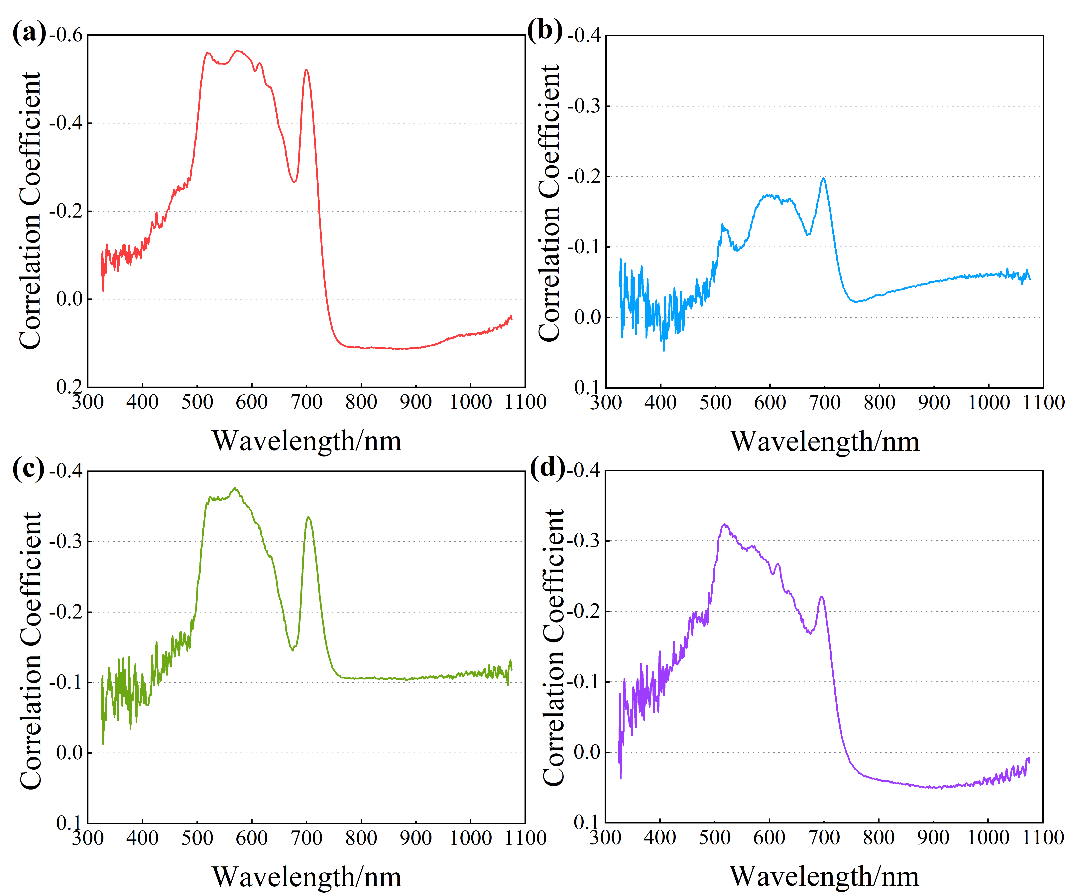


Figure S1. Correlation coefficients between LCC of upper layer, middle layer, lower layer, all LCC data and raw hyperspectral reflectance. (a-d) represented all LCC data, upper layer, middle layer and lower layer, respectively.
