# Supplementary material for: Combine photosynthetic characteristics and leaf hyperspectral reflectance for early detection of water stress
Source: Front Plant Sci. 2025 Apr 9;16:1520304. doi: 10.3389/fpls.2025.1520304 (PMC12014561; doi:10.3389/fpls.2025.1520304)
Supplement: Supplementary file 2 [file DataSheet2.docx]

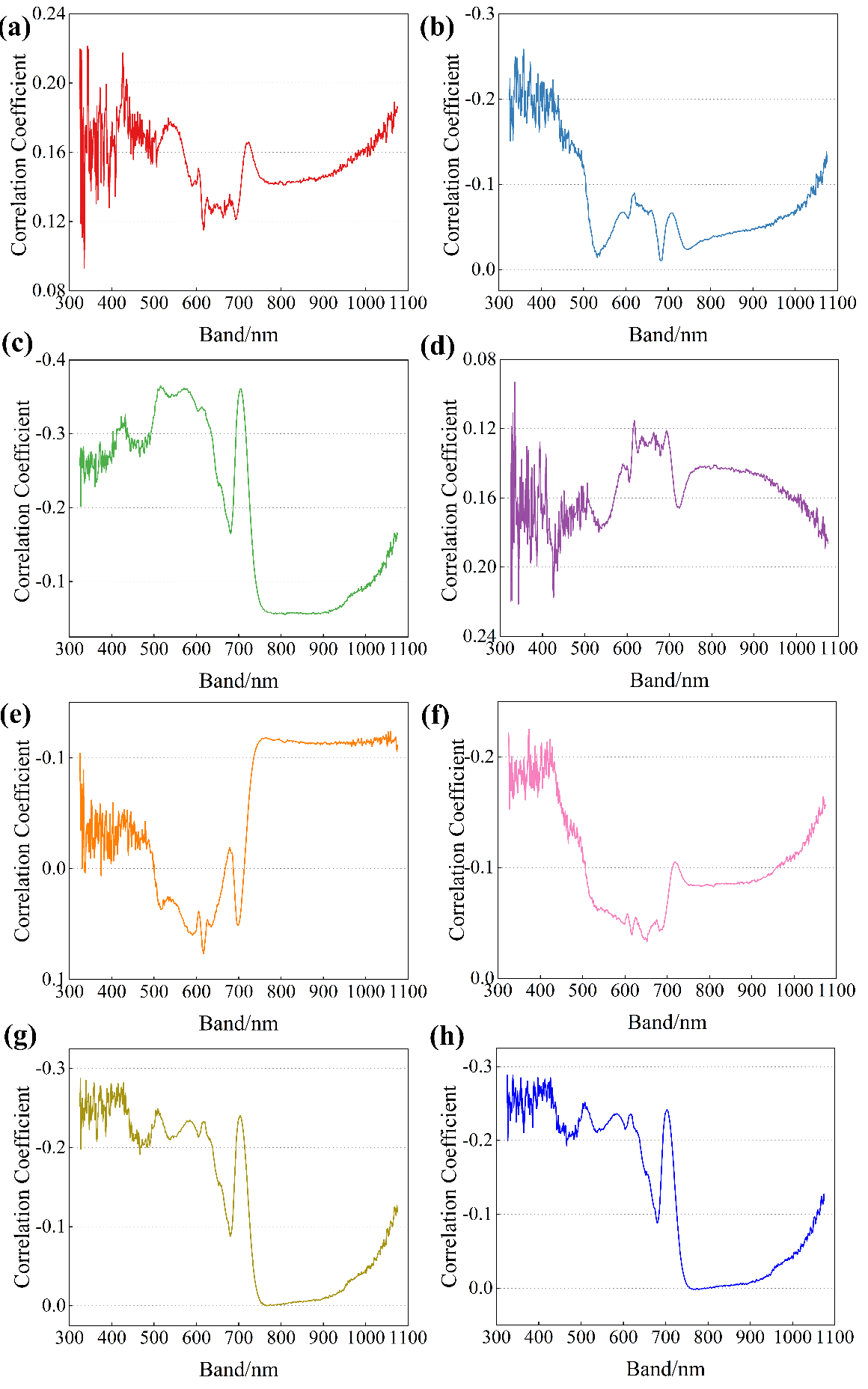


Figure S2. Correlation coefficients between physiological indicators and hyperspectral parameters. (a), (b), (c), (d), (e), (f), (g) and (h) indicated NPQ, Fv'/Fm', ETRmax, Fm’, qL, qP, Y(Ⅱ) and ERT respectively.
