# Supplementary material for: Combine photosynthetic characteristics and leaf hyperspectral reflectance for early detection of water stress
Source: Front Plant Sci. 2025 Apr 9;16:1520304. doi: 10.3389/fpls.2025.1520304 (PMC12014561; doi:10.3389/fpls.2025.1520304)
Supplement: Supplementary file 3 [file Table1.docx]

Supplemental Table S1 Performance of the hyperspectral SVIs for revealing the difference of drought treatments.

| DATE  VI | 3Day | 4Day | 5Day | 7Day | 8Day | 9Day | 10Day | 11Day | 12Day | 13Day | 15Day | 16Day | 17Day |
| --- | --- | --- | --- | --- | --- | --- | --- | --- | --- | --- | --- | --- | --- |
| **mSR_705** | 1.77 ± 0.32^a^ | 1.70 ± 0.26^ab^ | 1.69 ± 0.21^ab^ | 1.60± 0.27^bc^ | 1.61 ± 0.28^bc^ | 1.64 ± 0.23^bc^ | 1.55 ± 0.33^c^ | 1.48 ± 0.31^c^ | 1.52 ± 0.24^c^ | 1.52 ± 0.27^c^ | 1.6 ± 0.22^ab^ | 1.63 ± 0.23^ab^ | 1.65 ± 0.23^ab^ |
| **CAR_green** | 4.4 ± 1.03^a^ | 3.92 ± 0.82^ab^ | 3.86 ± 0.63^ab^ | 3.57 ± 0.63^bc^ | 3.53 ± 0.94^bc^ | 3.51 ± 0.74^bc^ | 3.37 ± 0.95^c^ | 3.26 ± 1.04^c^ | 3.23 ± 0.81^c^ | 3.63 ± 0.86^bc^ | 3.70 ± 0.62^bc^ | 3.90 ± 0.75^ab^ | 3.97 ± 0.79^ab^ |
| **SR(735/720)** | 1.27 ± 0.083^a^ | 1.26 ± 0.069^ab^ | 1.27 ± 0.059^a^ | 1.25 ± 0.08^ab^ | 1.23 ± 0.07^ab^ | 1.25 ± 0.061^ab^ | 1.22 ± 0.073^b^ | 1.23 ± 0.057^ab^ | 1.23 ± 0.056^b^ | 1.23 ± 0.066^ab^ | 1.25 ± 0.057^ab^ | 1.25 ± 0.054^ab^ | 1.26 ± 0.054^ab^ |
| **VOG1** | 1.31 ± 0.10^a^ | 1.3 ± 0.084^ab^ | 1.32 ± 0.072^a^ | 1.3 ± 0.097^ab^ | 1.28 ± 0.085^b^ | 1.29 ± 0.074^b^ | 1.26 ± 0.088^b^ | 1.27 ± 0.068^b^ | 1.27 ± 0.067^b^ | 1.27 ± 0.08^b^ | 1.29 ± 0.069^ab^ | 1.3 ± 0.065^ab^ | 1.31 ± 0.066^ab^ |
| **Datt1** | 0.535 ± 0.043^a^ | 0.52 ± 0.032^ab^ | 0.536 ± 0.02^a^ | 0.509 ± 0.037^bc^ | 0.495 ± 0.039^bc^ | 0.513 ± 0.033^bc^ | 0.478 ± 0.036^c^ | 0.495 ± 0.024^bc^ | 0.471 ± 0.031^c^ | 0.50 ± 0.032^ab^ | 0.508 ± 0.02^ab^ | 0.52 ± 0.031^ab^ | 0.526 ± 0.041^ab^ |
| **SR(750/710)** | 1.90 ± 0.21^a^ | 1.84 ± 0.25^ab^ | 1.89 ± 0.22^a^ | 1.83 ± 0.29^ab^ | 1.77 ± 0.26^ab^ | 1.75 ± 0.22^ab^ | 1.73 ± 0.27^b^ | 1.75 ± 0.21^b^ | 1.75 ± 0.20^b^ | 1.77 ± 0.24^ab^ | 1.81 ± 0.21^ab^ | 1.82 ± 0.2^ab^ | 1.87 ± 0.2^ab^ |
| **RENDVI** | 0.205 ± 0.055^a^ | 0.199 ± 0.046^ab^ | 0.207 ± 0.041^a^ | 0.194 ± 0.052^ab^ | 0.186 ± 0.049^ab^ | 0.196 ± 0.042^ab^ | 0.176 ± 0.049^b^ | 0.172 ± 0.038^b^ | 0.172 ± 0.039^b^ | 0.188 ± 0.048^ab^ | 0.189 ± 0.039^ab^ | 0.199 ± 0.037^ab^ | 0.199 ± 0.036^ab^ |
| **RE_NDVI** | 0.163 ± 0.046^a^ | 0.159 ± 0.039^ab^ | 0.165 ± 0.034^a^ | 0.156 ± 0.044^ab^ | 0.147 ± 0.04^ab^ | 0.156 ± 0.035^ab^ | 0.14 ± 0.041^b^ | 0.145 ± 0.032^ab^ | 0.144 ± 0.032^ab^ | 0.149 ± 0.039^ab^ | 0.151 ± 0.033^ab^ | 0.156 ± 0.031^ab^ | 0.159 ± 0.031^ab^ |
| **Carte4** | 0.532 ± 0.089^c^ | 0.545 ± 0.078^abc^ | 0.529 ± 0.064^c^ | 0.552 ± 0.082^abc^ | 0.57 ± 0.085^abc^ | 0.55 ± 0.07^abc^ | 0.584 ± 0.086^a^ | 0.573 ± 0.069^abc^ | 0.573 ± 0.07^ab^ | 0.568 ± 0.079^abc^ | 0.555 ± 0.066^abc^ | 0.548 ± 0.061^abc^ | 0.535 ± 0.061^bc^ |
| **SR(750/700)** | 3.46 ± 0.89^a^ | 3.32 ± 0.68^ab^ | 3.34 ± 0.59^ab^ | 3.19 ± 0.47^bc^ | 3.04 ± 0.7^bc^ | 3.07 ± 0.59^bc^ | 2.93 ± 0.75^c^ | 2.90 ± 0.6^c^ | 2.79 ± 0.56^c^ | 3.05 ± 0.68^bc^ | 3.13 ± 0.57^abc^ | 3.16 ± 0.54^abc^ | 3.31 ± 0.56^abc^ |
| **SR(752/690)** | 7.90 ± 0.50^a^ | 7.25 ± 0.70^b^ | 7.15 ± 0.45^b^ | 6.98 ± 0.50^b^ | 6.73 ± 0.50^b^ | 6.61 ± 0.70^c^ | 6.59 ± 0.70^c^ | 6.6 ± 0.60^c^ | 6.69 ± 0.80^c^ | 6.62 ± 0.50^c^ | 6.77 ± 0.60^b^ | 7.00 ± 0.7^ab^ | 7.24 ± 0.8^a^ |
| **Datt3** | 2.60 ± 0.58^a^ | 2.57 ± 0.25^ab^ | 2.56 ± 0.2^ab^ | 2.46 ± 0.33^bc^ | 2.45 ± 0.26^bc^ | 2.45 ± 0.2^bc^ | 2.27 ± 0.29^c^ | 2.32 ± 0.19^c^ | 2.31 ± 0.27^c^ | 2.36 ± 0.25^abc^ | 2.41 ± 0.28^bc^ | 2.44 ± 0.26^bc^ | 2.53 ± 0.27^ab^ |
| **NDVI705** | 0.405 ± 0.087^a^ | 0.389 ± 0.075^ab^ | 0.405 ± 0.062^a^ | 0.385 ± 0.079^ab^ | 0.367 ± 0.082^ab^ | 0.386 ± 0.068^ab^ | 0.353 ± 0.083^b^ | 0.364 ± 0.068^ab^ | 0.364 ± 0.067^ab^ | 0.369 ± 0.076^ab^ | 0.382 ± 0.063^ab^ | 0.387 ± 0.059^ab^ | 0.401 ± 0.059^a^ |
| **VOG2** | -0.0515 ± 0.019^c^ | -0.0484 ± 0.016^abc^ | -0.0512 ± 0.014^bc^ | -0.0482 ± 0.019^abc^ | -0.0445 ± 0.016^abc^ | -0.0471 ± 0.014^abc^ | -0.0423 ± 0.016^a^ | -0.0429 ± 0.012^ab^ | -0.0429 ± 0.012^ab^ | -0.0442 ± 0.015^abc^ | -0.0463 ± 0.013^abc^ | -0.0469 ± 0.012^abc^ | -0.048 ± 0.012^abc^ |
| **PERVI** | 1.53 ± 0.18^a^ | 1.51 ± 0.15^ab^ | 1.53 ± 0.13^a^ | 1.49 ± 0.17^ab^ | 1.47 ± 0.15^ab^ | 1.49 ± 0.13^ab^ | 1.44 ± 0.15^b^ | 1.45 ± 0.11^ab^ | 1.45 ± 0.11^ab^ | 1.47 ± 0.15^ab^ | 1.47 ± 0.12^ab^ | 1.5 ± 0.12^ab^ | 1.5 ± 0.11^ab^ |
| **Datt2** | 1.96 ± 0.34^a^ | 1.96 ± 0.27^ab^ | 1.95 ± 0.24^ab^ | 1.88 ± 0.22^bc^ | 1.82 ± 0.17^bc^ | 1.88 ± 0.24^bc^ | 1.78 ± 0.29^c^ | 1.80 ± 0.20^c^ | 1.80 ± 0.21^c^ | 1.78 ± 0.27^c^ | 1.85 ± 0.22^bc^ | 1.89 ± 0.21^bc^ | 1.91 ± 0.21^ab^ |
| **VOG3** | -0.12 ± 0.049^c^ | -0.112 ± 0.039^abc^ | -0.119 ± 0.035^bc^ | -0.111 ± 0.049^abc^ | -0.102 ± 0.039^abc^ | -0.108 ± 0.035^abc^ | -0.0963 ± 0.04^a^ | -0.0976 ± 0.03^ab^ | -0.0976 ± 0.03^a^ | -0.101 ± 0.038^abc^ | -0.106 ± 0.032^abc^ | -0.108 ± 0.031^abc^ | -0.111 ± 0.03^abc^ |
| **MTCI** | 1.39 ± 0.52^a^ | 1.31 ± 0.42^ab^ | 1.4 ± 0.38^a^ | 1.31 ± 0.53^ab^ | 1.20 ± 0.23^b^ | 1.18 ± 0.37^b^ | 1.12 ± 0.43^b^ | 1.16 ± 0.32^b^ | 1.15 ± 0.32^b^ | 1.19 ± 0.39^ab^ | 1.26 ± 0.35^ab^ | 1.27 ± 0.32^ab^ | 1.35 ± 0.32^ab^ |
| **TCARI** | 0.208 ± 0.061^d^ | 0.289 ± 0.097^a^ | 0.246 ± 0.06^bcd^ | 0.25 ± 0.069^bcd^ | 0.235 ± 0.069^cd^ | 0.239 ± 0.056^cd^ | 0.289 ± 0.078^a^ | 0.27 ± 0.057^abc^ | 0.262 ± 0.053^abc^ | 0.283 ± 0.09^ab^ | 0.248 ± 0.054^bcd^ | 0.23 ± 0.051^cd^ | 0.278 ± 0.05^ab^ |
| **NPCI** | -0.15 ± 0.089^ab^ | -0.157 ± 0.077^ab^ | -0.155 ± 0.077^ab^ | -0.182 ± 0.092^bc^ | -0.193 ± 0.082^bc^ | -0.198 ± 0.08^bc^ | -0.211 ± 0.078^c^ | -0.216 ± 0.058^c^ | -0.208 ± 0.063^c^ | -0.205 ± 0.065^c^ | -0.158 ± 0.062^ab^ | -0.165 ± 0.049^ab^ | -0.151 ± 0.053^ab^ |
| **OSAVI2** | 0.389 ± 0.076^b^ | 0.372 ± 0.068^ab^ | 0.383 ± 0.057^a^ | 0.361 ± 0.07^ab^ | 0.337 ± 0.072^b^ | 0.359 ± 0.06^ab^ | 0.334 ± 0.075^b^ | 0.344 ± 0.065^ab^ | 0.342 ± 0.061^b^ | 0.339 ± 0.07^b^ | 0.358 ± 0.057^ab^ | 0.356 ± 0.052^ab^ | 0.388 ± 0.058^a^ |
| **GNDVIhyper1** | 0.513 ± 0.082^a^ | 0.510 ± 0.034^a^ | 0.512 ± 0.022^a^ | 0.495 ± 0.032^b^ | 0.479 ± 0.038^b^ | 0.50 ± 0.029^b^ | 0.468 ± 0.041^c^ | 0.481 ± 0.024^b^ | 0.488 ± 0.036^b^ | 0.482 ± 0.044^b^ | 0.488 ± 0.028^b^ | 0.506 ± 0.025^a^ | 0.515 ± 0.028^a^ |
